# Hydroxycitric acid reconstructs damaged articular cartilages by modifying the metabolic cascade in chondrogenic cells

**DOI:** 10.1016/j.ocarto.2024.100564

**Published:** 2024-12-24

**Authors:** Yoshiyuki Mizushina, Liping Sun, Megumi Nishio, Sanae Nagata, Takeshi Kamakura, Masayuki Fukuda, Kousuke Tanaka, Junya Toguchida, Yonghui Jin

**Affiliations:** a Department of Regeneration Sciences and Engineering, Institute for Life and Medical Sciences, Kyoto University, 53 Shogoin-Kawahara-cho, Sakyo-Ku, Kyoto, 606-8507, Japan; b Central R & D Laboratory, Kobayashi Pharmaceutical Co., Ltd., 1-30-3 Toyokawa, Ibaraki, 567-0057, Japan; c Department of Fundamental Cell Technology, Center for iPS Cell Research and Application, Kyoto University, 53 Shogoin-Kawahara-cho, Sakyo-Ku, Kyoto, 606-8507, Japan; d Department of Orthopaedic Surgery, Graduate School of Medicine, Kyoto University, 53 Shogoin Kawahara-cho, Sakyo-Ku, Kyoto, 606-8507, Japan

**Keywords:** Hydroxycitric acid, Osteoarthritis, Chondrogenesis, ATP citrate lyase, Mitochondria, β-catenin

## Abstract

**Objective:**

Osteoarthritis, a degenerative joint disease, requires innovative therapies due to the limited ability of cartilage to regenerate. Since mesenchymal stem cells (MSCs) provide a cell source for chondrogenic cells, we hypothesize that chemicals capable of enhancing the chondrogenic potential of MSCs with transforming growth factor-beta (TGFβ) in vitro may similarly promote chondrogenesis in articular cartilage in vivo.

**Design:**

Chemical compounds that enhance the TGFβ signaling for chondrogenesis were investigated utilizing mesenchymal stem cells derived from human induced pluripotent stem cells. The mechanisms of action underlying the identified compound were explored in vitro, and its therapeutic effects were validated in vivo using a mouse model of exercise-induced osteoarthritis.

**Results:**

Hydroxycitric acid (HCA) emerged as the lead compound. In vitro, HCA effectively enhanced chondrogenesis by inhibiting ATP citrate lyase, inducing citrate and alpha-ketoglutarate (α-KG), while reducing cytosolic acetyl coenzyme A (Ac-CoA). This induction of α-KG promoted collagen prolyl-4-hydroxylase activity, boosting hydroxyproline production and matrix formation. The reduction of Ac-CoA attenuated the inhibitory effect of β-catenin on mitochondrial activity by diminishing its acetylation. In vivo, orally administered HCA accumulated in joint tissues of mice and histological examination demonstrated newly synthesized cartilage tissues in damaged area. Analysis of joint tissue extracts revealed a downregulation of Ac-CoA and an upregulation of citrate and α-KG, accompanied by a systemic increase in an anabolic biomarker.

**Conclusions:**

HCA demonstrates promise as an osteoarthritis therapy by enhancing chondrogenic differentiation. Its ability to modulate crucial metabolic pathways and facilitate cartilage repair suggests potential for clinical translation, addressing a critical need in the treatment of osteoarthritis.

## 1 Introduction

Osteoarthritis (OA) is a disease characterized by the destruction of articular cartilage structure and abnormal subchondral bone formation, which affects millions of individuals worldwide and causes a significant reduction in quality of life because of compromised joint mobility and pain [1]. Various factors contribute to the development of OA encompassing genetic, metabolic, biochemical, and biomechanical elements. Genome wide association studies identified several disease-associated single nucleotide polymorphisms, highlighting a genetic component in its etiology [2]. Anatomical factors, including joint shape and alignments plays a role in influencing mechanical stresses on chondrocytes [3]. Obesity directly affects the cartilage through excessive loading and indirectly through the production of systemic factors such as adipokine [4,5]. Injuries to joint components are a prevalent cause of OA in young adults [6], while aging stands out as the primary risk factor, inducing changes in various cellular and molecular features of chondrocytes, which cause the loss of proteostasis, mitochondrial dysfunction, and cellular senescence [7].

Current therapies for OA are symptomatic pain management in the early stages and surgical interventions in advanced cases. There are no disease-modifying OA drugs capable of halting or delaying disease progression [8]. The intra-articular injection of hyaluronic acid has been widely used; however, its recommendation level varies among different guidelines [8]. Several clinical trials of intra-articular treatments of cellular or non-cellular materials are currently underway [9]. Non-cellular treatments encompass the administration of serum albumin, growth factors, and inhibitors for pro-inflammatory cytokines [[10], [11], [12], [13]]. Cellular treatments include the administration of platelet rich plasma [14], bone marrow aspirate concentrate [15], stromal vascular fraction [16], and mesenchymal stem cells (MSCs) from various sources [17]. The mechanism of action of cellular treatments are not yet fully understood. Administrated cells may directly contribute to the regeneration of cartilage tissues by the differentiation into chondrocytes. Alternatively, they might accelerate the regenerative properties of intrinsic progenitors, such as MSCs in bone marrow or synovial tissues, by producing chemical mediators [18].

In vitro experiments with MSCs derived from various sources, including induced pluripotent stem cells (iPSCs), require the transforming growth factor-beta (TGFβ) signaling for the chondrogenic differentiation [19,20], and a combined treatment with the bone morphogenetic protein (BMP) signaling has been shown to promote MSCs differentiation [21]. The presence of TGFβ in OA joints fluid suggests a potential strategy for administrating compounds that enhance the chondrogenic properties of TGFβ in OA joints [22,23]. This approach aims to stimulate the intrinsic regenerative capability of progenitors such as MSCs.

Application of human bone marrow MSCs (BM-MSCs) for a large-scale screening encounters challenges due to the limited growth properties. To address this, we have established the method to induce MSCs from iPSCs via neural crest lineage. These iPSC-derived MSCs (iMSCs) exhibit chondrogenic properties and can be propagated to obtain enough cell quantity for the large-scale screening [24]. Utilizing these iMSCs, we conducted a drug screening for an intractable genetic disease and successfully identified a drug candidate [25].

Here, we conducted a screening of low-weight-molecular chemicals with the aim of enhancing the chondrogenic properties of TGFβ, utilizing human iMSCs and human BM-MSCs. Libraries consisting of naturally occurring chemicals were chosen for screening due to their potential clinical applicability. Our investigation identified hydroxycitric acid (HCA) as the most effective compound in enhancing the chondrogenic activity of TGFβ signal. Subsequent analyses of the mechanism of action unveiled that HCA promotes the chondrogenic differentiation of MSCs through at least two biochemical processes. Firstly, it promotes hydroxyproline formation via α-ketoglutarate/collagen prolyl 4-hydroxylase pathway. Additionally, HCA protects mitochondrial activity from the inhibitory effect of β-catenin. Oral administration of HCA to mice faithfully reproduced metabolic modifications observed in vitro, leading to the synthesis of cartilage tissues in damaged knee joint. These findings position HCA as a promising disease-modifying chemical for OA.

## 2 Materials and methods

### 2.1 Cell culture

Human iPSCs (414C2) [26] were maintained in primate embryonic stem cell medium (ReproCELL Inc., Tokyo, Japan) supplemented with 4 ​ng/mL recombinant human fibroblast growth factor-2 (FGF2) (R&D Systems, Minneapolis, MN) as previously described [27]. The induction and maintenance of iMSCs were conducted as previously described [24], maintained in αMEM medium with fetal bovine serum (FBS) and FGF2 (Supplementary Table S1A), and the first passage number (PN1) of iMSCs was used in this study.

Human bone marrow aspirates were obtained from patients undergoing autologous bone transplantation from iliac bones and bone marrow-derived MSCs was isolated and cultured in αMEM-GlutaMax with FBS and FGF2 (Supplementary Table S1B) as described previously [28]. Up to 4 passages were used in this study.

### 2.2 Two-dimensional chondrogenic induction (2D-CI)

2D-CI was conducted following a modified protocol based on the method previously described [29]. Briefly, PN1 iMSCs (1.0 ​× ​10^5^ ​cells) were suspended in 100 ​μL of chondrogenic basal medium (Supplementary Table S2A) and then transferred to Matrigel-coated-96-well plates (Corning Inc., Corning, NY). After 24 ​h, 150 ​μL of the chondrogenic basal medium supplemented with appropriate reagents (Supplementary Table S2B) was added. Cultures were maintained at 37 ​°C under 5 ​% CO_2_ for 12 days, and the culture medium was refreshed every 3 days.

### 2.3 Three-dimensional chondrogenic induction (3D-CI)

3D-CI was conducted following a modified protocol based on methods previously described [20,28]. Briefly, PN1 iMSCs or PN4 BM-MSCs (2.5 ​× ​10^5^ ​cells each) were suspended in 0.5 ​mL of chondrogenic basal medium supplemented with appropriate reagents (Supplementary Tables S2A and B) and then transferred to 15 ​mL tubes (Corning Inc.). The cells were centrifuged at 1000 ​rpm for 3 ​min to form pellets and maintained at 37 ​°C under 5 ​% CO_2_ for 21 days. The culture medium was refreshed every 3 days.

### 2.4 Alcian Blue (AB) staining

Briefly, induced 2D cells were fixed for 30 ​min with 4 ​% paraformaldehyde (Fujifilm Wako Pure Chemical Corp., Osaka, Japan) and rinsed with phosphate buffered saline (PBS). Subsequently, these cells were then stained for 1 ​h with AB solution (1 ​% AB, pH 1) (Muto Pure Chemical Co., Ltd, Tokyo, Japan), followed by three washes with PBS. For quantification of the staining, AB dye was dissolved by adding 150 ​μL of Dissociation Reagent (Biocolor Ltd., Belfast, UK) to each well of a 96-well plate, and then measured at 620 ​nm using a SpectraMax M2RF plate reader (Molecular Devices LLC, San Jose, CA).

### 2.5 Glycosaminoglycan (GAG) and double-stranded DNA (dsDNA) quantification

The 2D-CI cultured monolayers and the 3D-CI pellets were washed twice with PBS, and lysed with papain buffer. The papain buffer consisted of 2 ​mg/mL papain (Sigma-Aldrich, St. Louis, MO), 5 ​mM l-cysteine hydrochloride monohydrate and 10 ​mM EDTA in PBS. The resulting buffer solutions were transferred to microtubes and incubated at 60 ​°C for 6 ​h while mixing at 2000 ​rpm (Eppendorf ThermoMixer C; Eppendorf, Hamburg, Germany). The GAG content in the lysed solution was quantified with the Blyscan Dye & Dissociation Reagents kit (Biocolor Ltd.) and measured by absorbance intensity at 656 ​nm using a SpectraMax M2RF plate reader (Molecular Devices LLC) in accordance with the manufacturer's protocols.

The DNA content of the lysed solution was quantified using the PicoGreen dsDNA Quantitation Kit (Invitrogen Corp., Carlsbad, CA). Measurement of fluorescence intensity (λex ​= ​480 ​nm/λem ​= ​520 ​nm) was carried out using a SpectraMax M2RF plate reader (Molecular Devices LLC) in accordance with the manufacturer's protocols.

### 2.6 Reverse transcription quantitative polymerase chain reaction (RT-qPCR) analysis

Briefly, 4.0 ​× ​10^5^ ​cells per condition were plated in 24-well plate, cultured and washed twice with PBS, and then lysed with RLT buffer (Qiagen Inc., Valencia, CA). Total RNA was purified with the RNeasy Mini Kit (Qiagen Inc) and genomic DNA was eliminated with the DNase-one Kit (Qiagen Inc). Subsequently 0.3 ​μg of total RNA was reverse transcribed for single-stranded cDNA using ReverTra Ace qPCR RT Master Mix (TOYOBO Co., Ltd., Osaka, Japan), following the manufacturer's instructions. Quantitative PCR was performed with Thunderbird SYBR qPCR Mix (TOYOBO) and analyzed with the StepOne real-time PCR system (Applied Biosystems, Waltham, MA). Primers used are listed in Supplementary Table S3A.

### 2.7 Histological analysis of chondrocyte pellets

3D chondrocyte pellets were transferred to microtubes, washed with PBS and fixed with 4 ​% paraformaldehyde overnight at 4 ​°C. Fixed pellets were embedded in paraffin and sectioned using a microtome. The sections were deparaffinized and stained with Hematoxylin-Eosin (HE), AB or Safranin O. Imaging the stained sections was conducted using an All-in-One Fluorescence Microscope BZ-X800 (KEYENCE Corp., Osaka, Japan), and the Safranin O-positive area in each section was quantified using the ImageJ software.

### 2.8 siRNA transfection

iMSCs of PN1 were grown to 60 ​%–70 ​% confluency in αMEM media with 10 ​% FBS supplement. Appropriate amounts of siRNAs (Supplementary Table S3B) were diluted in Opti-MEM media following the manufacturer's instructions. The diluted siRNA and Lipofectamine RNAiMAX transfection Reagent (Thermo Fisher Scientific Inc., Waltham, MA) were combined and incubated at room temperature for 10 ​min, and then the resulting siRNA complex media were added into the iMSC. After 12 ​h of incubation at 37 ​°C in 5 ​% CO_2_, the transfected iMSCs (4.0 ​× ​10^5^ ​cells) were transferred to 24-well plate (Corning Inc) pre-coated with Matrigel and cultured in chondrogenic basal media supplemented with appropriate reagents (Supplementary Table S2B) for 12 days at 37 ​°C in 5 ​% CO_2_ incubator. The culture media were refreshed every 3 days.

### 2.9 Western blotting

iMSCs were seeded at 5.0 ​× ​10^6^ per 6 ​cm dish (Corning Inc) precoated with Matrigel and cultured for 2D-CI. Total cell lysates were prepared using RIPA buffer with protease inhibitor cocktails (Nacalai Tesque Inc., Kyoto, Japan). The protein concentration of cell lysate sample was measured using a Pierce BCA Protein Assay Kit (23,225; Thermo Fisher Scientific) with a SpectraMax M2RF plate reader (Molecular Devices LLC). Proteins (20 ​μg) were separated by 7.5 ​% SDS-PAGE and transferred onto PVDF membranes (Bio-Rad Laboratories, Inc., Hercules, CA). Antibodies were diluted with Antibody diluent solution 1 or 2 (NKB-101, TOYOBO) to appropriate concentration for each antibody (Supplementary Table S4). Immunoprecipitation of SOX9 protein was performed using a Dynabeads Protein G Immunoprecipitation Kit (10007D; Thermo Fisher Scientific) following the manufacturer's protocol. Protein bands were detected with ECL Prime Western Blotting Detection Reagent (RPN2232; GE Healthcare, Little Chalfont, UK), and visualization and quantification were performed using ChemiDoc Touch MP imaging system with Image Lab software (Bio-Rad Laboratories, Inc.).

### 2.10 Fractionation of cytoplasmic and mitochondrial fractions and metabolites analysis

iMSCs of PN1 (>1.0 ​× ​10^7^ ​cells) were suspended in 30 ​mL of chondrogenic basal medium, and subsequently transferred to two 15 ​cm dishes (BD Biosciences, Franklin Lakes, NJ) precoated with matrigel. After 24 ​h, a total of 15 ​mL of the chondrogenic basal medium supplemented with 20 ​ng/mL Activin A (ActA) and/or 100 ​μM HCA was added. Monolayer cultures were maintained at 37 ​°C under 5 ​% CO_2_ for 3 or 6 days. Following culture, cells were collected, counted, and precisely prepared to reach 1.0 ​× ​10^7^ ​cells on ice. The cells were fractionated into cytoplasmic and mitochondrial fractions by using a special kit (Mitochondria isolation kit for cultured cells, 89,874; Thermo Fisher Scientific) following the manufacturer's protocol. Acetyl coenzyme A (Ac-CoA), citric acid and α-KG in these fractions were detected using their corresponding assay kits: MAK039, MAK057, and MAK054 (from Sigma-Aldrich) and quantified by fluorescence intensity (λex ​= ​535 ​nm/λem ​= ​587 ​nm) using a SpectraMax M2RF plate reader (Molecular Devices LLC).

### 2.11 Hydroxyproline (Hyp) measurement

The 4-hydroxyproline (4-Hyp) concentration was determined using Hydroxyproline assay kit (STA-675; Cell Biolabs Inc., San Diego, CA, USA), employing colorimetric ELISA. iMSCs were seeded at 2.0 ​× ​10^6^ per well of 6-well plate (Corning Inc) and cultured for 6 days. Following cultivation, cells were collected, counted, and prepared to a precise 1.5 ​× ​10^6^. After two washes with PBS, transferred to glass vial containing 1 ​mL 5 ​N HCl, and subsequently incubated at 120 ​°C for 3 ​h for hydrolyzation. The resulting acid-hydrolyzed product underwent filtration through a 0.45 ​μm PVDF syringe filter (Sigma-Aldrich) and was left to dry under vacuum for 45 ​min at 70 ​°C. The hydroxyproline assay kit was then employed for the analysis, measuring absorbance at 540 ​nm after addition of chloramine T and Ehrlich's reagent.

### 2.12 Measurements of mitochondrial oxygen consumption rate (OCR)

Mitochondrial function in iMSCs, cultured with chondrogenic basal medium for 6 days, was assessed in real-time using the Seahorse XF Cell Mito Stress Test kit (Agilent Technologies, Santa Clara, CA) on Seahorse XF96 Extracellular Flux Analyzer (Agilent Technologies). Briefly, chondrogenic differentiated cells were resuspended by the treatment with Accutase (Thermo Fisher Scientific), filtered by 70-μm cell strainers, and reseeded (5.0 ​× ​10^4^/well) into a Matrigel-precoated seahorse 96-well mini plate. After achieving uniform adhesion, the media were changed with XF Assay Medium supplemented with 10 ​mM d-glucose, 2 ​mM l-glutamine, and 1 ​mM pyruvate (pH 7.4) OCR was measured initially at baseline, followed by sequential injections of oligomycin (4 ​μM), trifluoromethoxy carbonylcyanide phenylhydrazone (FCCP) (2 ​μM), and rotenone/antimycin A (1 ​μM). The basal respiration, maximal respiration, and ATP production were calculated according to the manufacturer's manual.

Basal respiration refers to oxygen consumption used to meet the cellular ATP demand resulting from mitochondrial proton leak, indicating the energetic demand of the cell under baseline condition. The decrease in OCR upon injection of oligomycin, an ATP synthase inhibitor, represents the portion of basal respiration dedicating to driving ATP production. This demonstrates the ATP produced by the mitochondria to fulfilling the energetic needs of the cell. Maximal respiration represents the peak OCR attained by adding the uncoupler FCCP. FCCP stimulates the respiratory chain to operate at maximum capacity by mimicking a physiological “energy demand”. Hence, the maximal respiration reflects the highest rate of respiration that the cell can achieve.

### 2.13 Measurement of mitochondrial membrane potential

iMSCs were cultured in chondrogenic basic medium with appropriate reagents for 6 days. Subsequently, the culture cells were reseeded at 2.0 ​× ​10^5^/well in 12-well plate (Corning Inc.) and further cultured with chondrogenic basal medium for 12 ​h. The assessment of mitochondrial membrane potential was conducted using the potential-dependent fluorescent dye tetramethylrhodamine ethyl ester (TMRE). The 12-well plate was loaded with TMRE and Hoechst33342 for 30 ​min and 10 ​min, respectively, at 37 ​°C in the dark. Following incubation, cells were washed with chondrogenic basal medium and then observed and imaged using an All-in-One Fluorescence Microscope BZ-X800 (KEYENCE Corp.). Typically, data from 50 to 60 ​cells were collected for each experimental condition. From the obtained image data, TMRE signal was quantified using ImageJ software, and Hoechst33342 stained cells were counted.

### 2.14 Mouse model

Male mice (C57Bl/6; Japan SLC, Inc., Hamamatsu, Japan) aged 12 weeks were used for the in vivo experiments. The mice were housed in a controlled environment with a relative humidity of 50 ​± ​10 ​% and a temperature of 25 ​± ​2 ​°C on a 12 ​h dark-light cycle. Mice were either maintained at cage activity until sacrifice or were subjected to enforced running for 2 weeks on a MK-690/RM treadmill (Muromachi Kikai Co., Ltd., Tokyo, Japan) for 20 ​min/day, at 20 ​m/min and an uphill elevation of 15° as described [30]. After two weeks of forced running, mice were randomly assigned to two groups (*n* ​= ​5 for each group). In determining the sample size, our choice of animal number was guided by the dual objectives of achieving statistically significant results and adhering to ethical standards. The control group was administered orally with distilled water, while the experimental group was administered orally with 308 ​mg/kg/day HCA dissolved in distilled water for a period of 8 weeks. The dosage of administrated HCA was determined by the human-mouse conversion equation [31], using the maximum daily uptake of HCA (1,500mg/60 ​kg/day) recommended by Pharmaceutical and Medical Devices Agency of Japan.

### 2.15 Histological analysis and qualitative scoring of articular cartilage

Mice were euthanized by CO_2._ For histopathological specimen staining of articular cartilage, the femur of the knee joint of the right leg served as the source. The femurs were meticulously dissected with removal of adherent soft tissue, followed by a brief wash in PBS treated with proteinase inhibitor. Subsequently, the bones were fixed in 10 ​% formalin for a duration of 2 days. After fixation, the samples underwent decalcification in 10 ​% EDTA solution, followed by dehydration and embedding in paraffin. Focusing on the articular cartilage of the medial condyle of the femur, approximately 50 continuous 3 ​μm sections were vertically cut from the medial side and mounted on silane-coated slides to prepare the specimens. Representative sections of each specimen were stained with HE and Safranin O for subsequent histological scoring. The stained tissues were viewed using a BZ-X800 microscope (KEYENCE Corp.). In a blinded fashion, two examiners evaluated the histological condition of the articular cartilage surface and scored them utilizing the OARSI histopathology initiative (mouse OA scoring) system [32], using a score of 0 represents normal cartilage, 0.5 ​= ​loss of proteoglycan with an intact surface, 1 ​= ​superficial fibrillation without loss of cartilage, 2 ​= ​vertical clefts and loss of surface lamina (any % or joint surface area), 3 ​= ​vertical clefts/erosion to the calcified layer lesion for 1–25 ​% of the quadrant width, 4 ​= ​lesion reaches the calcified cartilage for 25–50 ​% of the quadrant width, 5 ​= ​lesion reaches the calcified cartilage for 50–75 ​% of the quadrant width, 6 ​= ​lesion reaches the calcified cartilage for >75 ​% of the quadrant width.

### 2.16 Measurement of cartilage metabolic markers in mice serum

Blood was collected when the mice were sacrificed, and serum was prepared following the manufacturer's protocols, then stored at −80 ​°C until use. Focusing on changes in cartilage metabolism, the serum samples were analyzed for collagen II synthesis biomarker (*c*-propeptide of type II procollagen, CPII) and degradation biomarker (collagen Type II cleavage, C2C). Serum CPII and C2C were measured using commercially available kit: CPII ELISA collagen type II synthesis assay (60-1003-001; IBEX Pharmaceuticals Inc., Quebec, Canada) and C2C ELISA collagen type II cleavage assay (60-1001-001; IBEX Pharmaceuticals Inc.), respectively, following the manufacturer's protocols. Colorimetric quantitation at 450 ​nm in ELISA was performed using a spectrophotometer (SpectraMax M2RF, Molecular Devices LLC).

### 2.17 Quantitative analysis of HCA and metabolites in mice knee joint tissues

One hour after oral administration of 308 ​mg/kg/day HCA, after the disinfection with 70 ​% ethanol, knee joints were exposed and removed by cutting femurs and tibiae. Subsequently, joint capsules were resected, and other tissues were kept on ice. These tissues, at approximately 150 mg/animal, were washed with saline, transferred to microtubes, and rapidly frozen in liquid nitrogen for 5 ​min. The frozen tissues were homogenized with the BM3000 Multi-beads shocker (Yasui Kikai Corporation, Osaka, Japan) following the manufacturer's instructions. The homogenized samples were transferred to 2 ​mL volume polystyrene microtubes containing 80 ​% methanol. Extraction was performed by sonication for 10 ​min on ice, followed by centrifugation (15,000 ​rpm, 15 ​min, 4 ​°C). The centrifuged supernatant was dried by evaporation, 250 ​μL of distilled water was added, and then the final extract was prepared by filtration through a 0.22 ​μm filter. HCA amount in the final extract was analyzed using liquid chromatography - mass spectrometry (LC-MS/MS) [33]. The metabolites in the final extract were quantified by fluorescence intensity (λex ​= ​535 ​nm/λem ​= ​587 ​nm) by a SpectraMax M2RF plate reader (Molecular Devices LLC) using their specialized kits according to the manufacturer's protocols.

### 2.18 Statistical analysis

Statistical analyses and graph generations were performed using Prism v9.4.1 (GraphPad Software Inc., La Jolla, CA). Data are presented as mean ​± ​SD unless specified otherwise in each figure. Statistical significance was determined by Student's *t*-test or a one-way or two-way ANOVA with multiple comparisons test as specified in each figure. p-values <0.05 were considered significant. Exact p-values are represented as 0.0001 ​< ​p ​< ​0.1.

### 2.19 Study approval

Experiments with human MSCs were performed with written informed consent and approved by the Ethics Committee of the Department of Medicine and Graduate School of Medicine, Kyoto University. Animal experiments were approved by the institutional animal committee of Kobayashi Pharmaceutical Co., Ltd., and performed in strict accordance with the Regulation on Animal Experimentation at Kobayashi Pharmaceutical Co., Ltd. as well as the principles of the Declaration of Helsinki.

### 2.20 Data availability

The data that support the findings of this study are available from the corresponding author upon reasonable request.

## 3 Results

### 3.1 Screening of compounds promoting chondrogenic differentiation of MSCs

The first screening was performed by the two-dimensional chondrogenic induction (2D-CI) of iMSCs employing 1000 naturally occurring low-molecular weight compounds (200 ​μM) consisting of 520 compounds from Sigma-Aldrich Co. LLC (St. Louis, MO) and 480 compounds from GreenPharma S.A.S. (Orléans, France). The evaluation was based on the formation of AB-positive matrix (Supplementary Fig. 1). Among these compounds, 12 showed the absorbance of AB more than 1.25 times higher than the control that was treated with ActA alone (Supplementary Fig. 1). The second screening was performed by three-dimensional chondrogenic differentiation (3D-CI) of human BM-MSCs with the selected 12 compounds (100 ​μM). Evaluation was based on the Safranin O-positive area in the maximum section of each cell pellet, which finally identified (−)-hydroxycitric acid (HCA) as the most effective compound (Supplementary Fig. 1).

### 3.2 HCA promoted the chondrogenic differentiation of MSCs

**Fig. 1:**
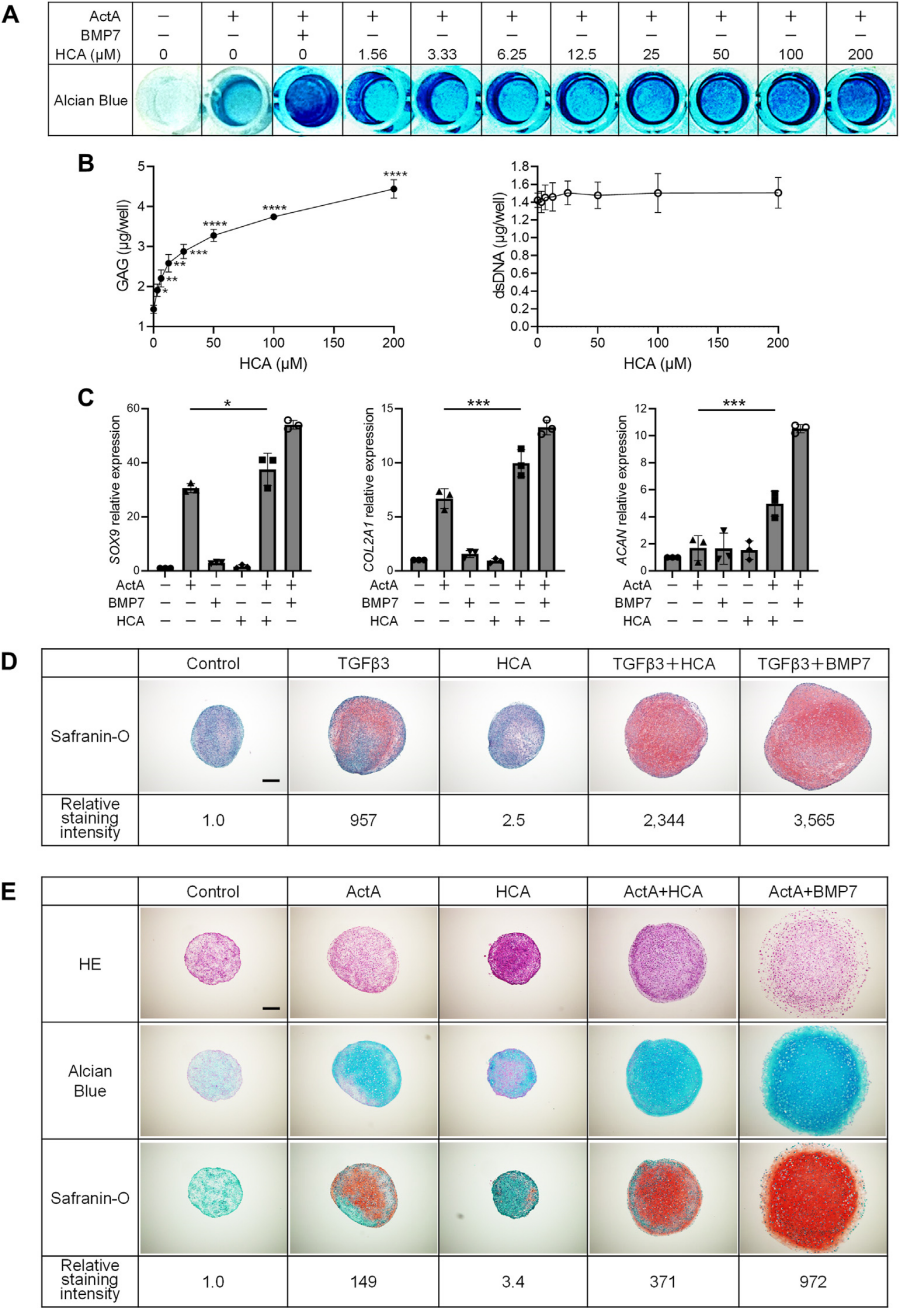
**Hydroxycitric acid (HCA) promoted the chondrogenic differentiation of MSCs. (A**–**C)** two-dimensional chondrogenic induction (2D-CI) of iPSCs-derived MSCs (iMSCs). Cells were cultured for 12 days using 96-well plates with chondrogenic basal medium containing Activin A (ActA) (20 ​ng/mL) and HCA at concentration as indicated. The assessment was conducted by AB staining **(A)**, quantification of glycosaminoglycan (GAG) and double stranded DNA (dsDNA) **(B)**, and measurement of mRNA expression of chondrocyte-related genes **(C)**. Cells treated with ActA (20 ​ng/mL) and BMP 7 (BMP7) (20 ​ng/mL) was used as a positive control in **(A)**. RNAs were extracted and assessed by reverse transcription quantitative polymerase chain reaction (RT-qPCR), and the expression level was normalized against samples cultured without ActA, BMP7 or HCA. **(D**–**E)** three-dimensional chondrogenic induction (3D-CI) of bone marrow-derived MSCs **(D)** and iMSC **(E)**. Cells were cultured in chondrogenic basal medium containing transforming growth factor-beta 3 (TGFβ3) (5 ​ng/mL), BMP7 (20 ​ng/mL) and/or HCA (100 ​μM) **(D)** or ActA (20 ​ng/mL), BMP7 (20 ​ng/mL) and/or HCA (100 ​μM) ​**(E)** in 3D condition for 21 days, and subsequently pellets were stained with Safranin O **(D)**, or Hematoxylin-Eosin (HE), AB or Safranin O **(E)**. Relative staining intensity of each section was demonstrated as the ratio of Safranin O positive area in each sample compared to that in samples cultured solely in basal medium (control). Scale bar indicates 200 ​μm. Statistical analysis was performed by Student's *t*-test compared to samples treated solely with ActA **(B)** or one-way ANOVA **(C)**. ∗*P* ​< ​0.05, ∗∗*P* ​< ​0.01, ∗∗∗*P* ​< ​0.001 and ∗∗∗∗*P* ​< ​0.0001. Values are mean ​± ​SD. *n* ​= ​3, independent experiments.

To validate the impact of HCA for the chondrogenic differentiation, several additional experiments were conducted. In a dose-dependent manner, HCA facilitated the formation of AB-positive matrix during 2D-CI of iMSC (Fig. 1A). Concurrently, the amount of glycosaminoglycan (GAG) exhibited a dose-dependent increase, while the number of cells, as indicated by the amount of dsDNA, showed no discernible difference (Fig. 1B), indicating that the augmented matrix formation was not attributed to proliferative effect of HCA on cell growth. Furthermore, the treatment with HCA resulted in the upregulation of mRNA expression of chondrogenic genes (Fig. 1C). It is noteworthy that this promotional effect was observed exclusively when HCA was co-administered with ActA.

Consistent with the screening outcome, the inclusion of HCA in 3D-CI of bone marrow-derived MSCs with TGFβ3 exhibited more intense Safranin O staining compared to samples treated with TGFβ3 alone (Fig. 1D). The influence of HCA on 3D-CI was further investigated using iMSC with ActA instead of TGFβ3. The combination of ActA and HCA resulted in markedly stronger AB and Safranin O staining compared to samples treated solely with ActA. These outcomes were comparable to those observed with the combination of ActA and bone morphogenetic protein 7 (BMP7) (Fig. 1E). It is crucial to emphasize that HCA alone showed little effect on 3D-CI.

In summary, HCA augmented the effect of TGFβ signaling for the chondrogenic induction of iMSC or BM-MSC, suggesting that HCA replaced the role of BMP signaling. However, treatments with HCA exhibited no impact on the induction of phosphorylated SMAD1/5 (Supplementary Fig. 2), suggesting that HCA may replace the role of BMPs in the chondrogenic induction through mechanisms independent of the BMP/SMAD signaling pathway.

### 3.3 ACLY inhibition promoted the chondrogenic differentiation of iMSC in 2D-CI

**Fig. 2:**
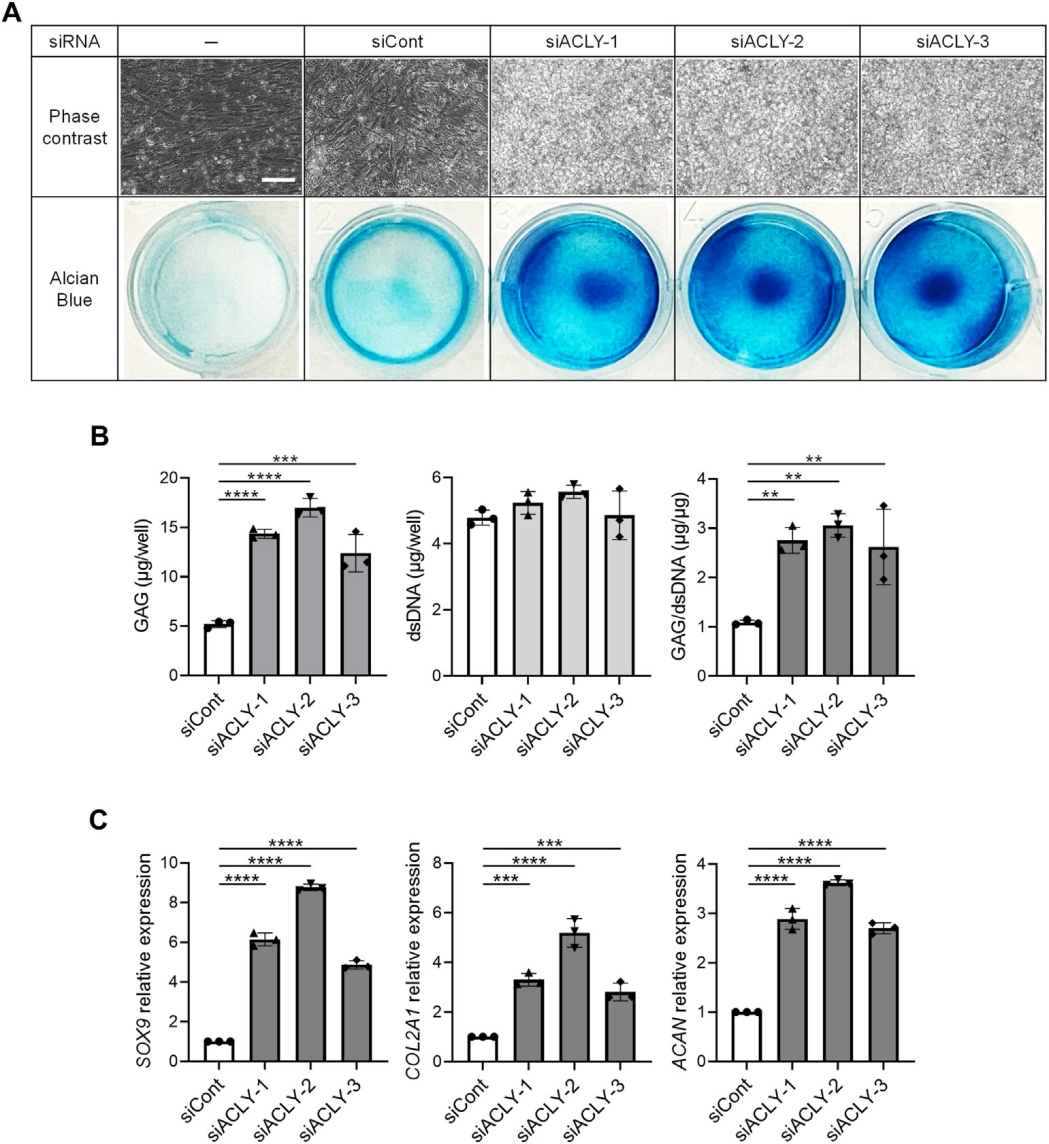
**Adenosine 5′-tri-phosphate citrate lyase (ACLY) inhibition promoted the chondrogenic differentiation of iPSCs-derived mesenchymal stem cells (iMSCs) in two-dimensional chondrogenic induction (2D-CI).** iMSCs were transfected with indicated siRNAs at the day before culture initiation and then cultured in chondrogenic basal medium with Activin A (ActA) (20 ​ng/mL) for 12 days. The assessment was conducted by phase contrast images and AB staining **(A)**, quantification of glycosaminoglycan (GAG) and double stranded DNA (dsDNA) **(B)**, and measurement of relative mRNA expression of chondrocyte-related genes **(C)**. The expression level was normalized against samples cultured with siRNA negative control (siCont). Scale bar ​= ​100 ​μm. Statistical significance was determined using one-way ANOVA **(B–C)**. ∗∗*P* ​< ​0.01, ∗∗∗*P* ​< ​0.001 and ∗∗∗∗*P* ​< ​0.0001). Values are mean ​± ​SD. *n* ​= ​3, independent experiments.

HCA functions as a competitive inhibitor of adenosine 5′-tri-phosphate citrate lyase (ACLY) [34]. To establish that the inhibition of ACLY by HCA is causative for the induction of chondrogenesis with TGFβ signaling, the expression of *ACLY* was inhibited by siRNA during 2D-CI. All three siRNAs against *ACLY* gene, which exhibited a constant suppressing effect on *ACLY* expression (Supplementary Fig. 3A), significantly enhanced the formation of AB-positive matrix in 2D-CI (Fig. 2A). This enhancement was accompanied by an increase in the amount of GAG without affecting the cell growth (Fig. 2B). Furthermore, mRNA expression of chondrogenesis-related genes was upregulated (Fig. 2C), providing the evidence that inhibition of ACLY effectively enhanced the 2D-CI. Consequently, it can be concluded that observed effect of HCA is attributed to the inhibition of ACLY.

### 3.4 HCA played a role of ACLY inhibitor and regulatory module of metabolites during 2D-CI

**Fig. 3:**
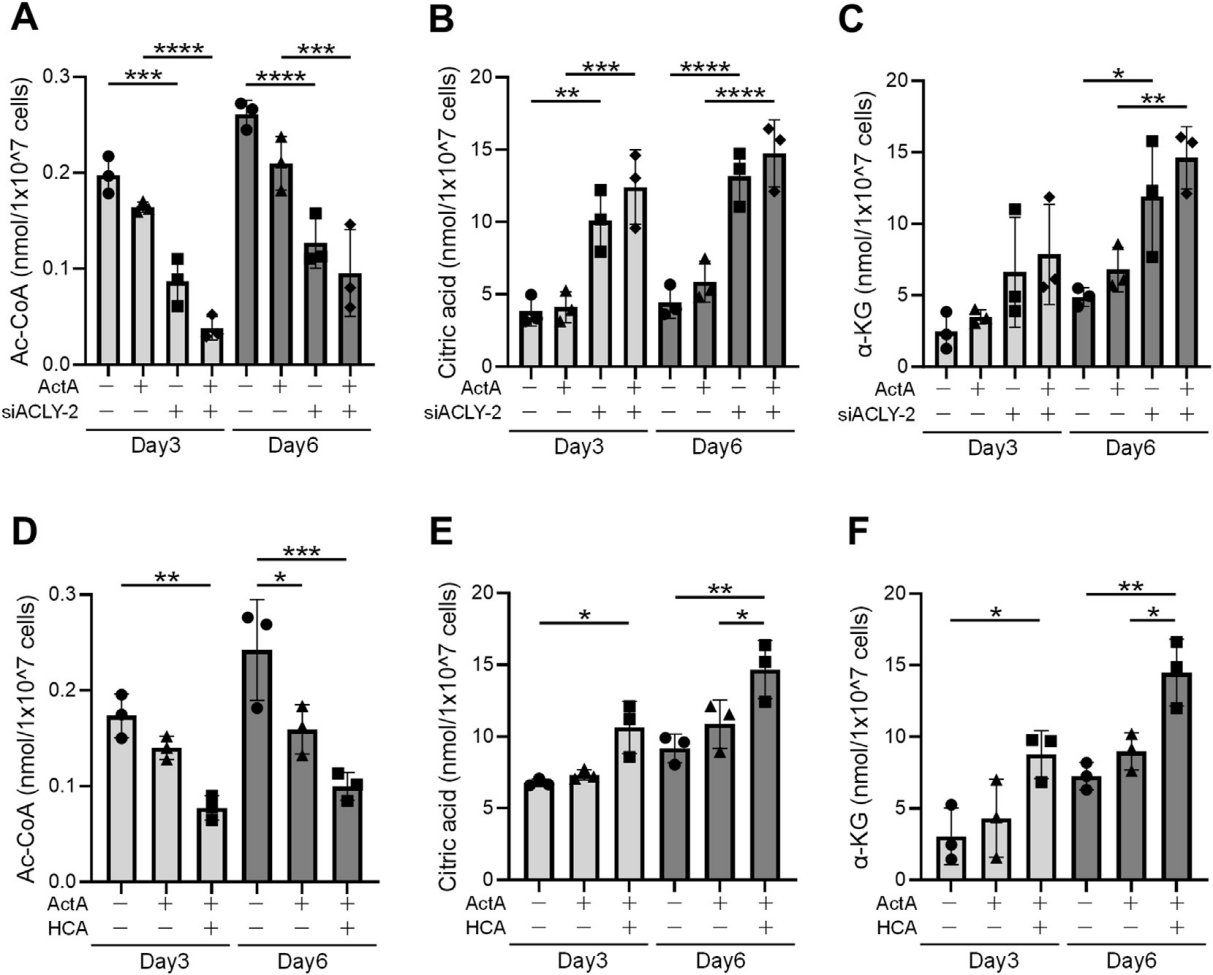
**Hydroxycitric acid (HCA) played a role of adenosine 5′-tri-phosphate citrate lyase (ACLY) inhibitor and regulatory module of metabolites during two-dimensional chondrogenic induction (2D-CI). (A**–**C)** Effect of ACLY knock-down on metabolites. iPSCs-derived mesenchymal stem cells (iMSCs) were cultured in chondrogenic basal medium with or without Activin A (ActA) (20 ​ng/mL) for 3 or 6 days. siRNA against *ACLY* gene (siACLY-2) was transfected at the day before culture initiation. Cytoplasmic fraction was extracted at the indicated day and the amount of acetyl coenzyme A (Ac-CoA) **(A)**, citric acid **(B)**, and alpha-ketoglutarate (α-KG) **(C)** was quantified. **(D**–**F)** Effect of HCA on metabolites. iMSCs were cultured in chondrogenic basal medium with ActA (20 ​ng/mL) and/or HCA (100 ​μM) for 3 or 6 days. Cytoplasmic fraction was extracted at the indicated day and the amount of Ac-CoA **(D)**, citric acid **(E)**, and α-KG **(F)** was quantified. Statistical significance was determined using one-way ANOVA (∗*P* ​< ​0.05, ∗∗*P* ​< ​0.01, ∗∗∗*P* ​< ​0.001 and ∗∗∗∗*P* ​< ​0.0001). Values are mean ​± ​SD. *n* ​= ​3, independent experiments.

ACLY is a major enzyme responsible for the synthesis of cytosolic acetyle coenzyme A (Ac-CoA), catalyzing the conversion of citrate and CoA to Ac-CoA and oxaloacetate [35]. To validate the inhibitory effect of HCA on ACLY in the context of 2D-CI, the level of Ac-CoA, citrate, and α-ketoglutarate (α-KG), the latter being synthesized from citrate through isocitrate, were compared in cells treated with HCA and siRNAs of siACLY-2 targeting the *ACLY* gene. Cell extracts were isolated from 2D-CI monolayers at day 3 or day 6 and subsequently fractionated into the cytoplasmic and mitochondrial fractions (Supplementary Fig. 4), and the amounts of metabolites in the cytoplasmic fraction was evaluated (Fig. 3). Downregulation of the *ACLY* gene alone resulted in a reduction in the amount of Ac-CoA, and this effect was further enhanced in the presence of Activin A (Fig. 3A). Simultaneously, the inhibition of *ACLY* gene led to an increase in the citrate level, with a more pronounced effect observed upon the addition of Activin A (Fig. 3B). These results were clearly observed even at day 3. In the case of α-KG, the impact of ACLY inhibition became significant by day 6, and the levels of α-KG were further augmented with the addition of Activin A (Fig. 3C). This temporal delay aligns with the expected metabolic progression from ACLY to α-KG. Treatment with HCA yielded nearly identical results for these metabolites, with the effect more pronounced at day 6. HCA treatment augmented the ActA-induced decrease in Ac-CoA (Fig. 3D), as well as the increase of citrate (Fig. 3E) and α-KG (Fig. 3F) in the cytoplasmic fraction. These results strongly indicated that HCA functions as an ACLY inhibitor during 2D-CI and thereby modifies metabolites.

### 3.5 HCA promoted the chondrogenic differentiation of iMSCs in 2D-CI via the α-KG – collagen prolyl 4-hydroxylase (CP4H) pathway

ACLY inhibition by either HCA or siRNA targeting *ACLY* gene resulted in an increase of cytoplasmic α-KG (Fig. 3C and F). α-KG plays a role in various biological activity, serving as an intermediate in the tricarboxylic acid cycle, precursor for amino acid synthesis, a participant in nitrogen metabolism, and a cosubstrate for α-KG-dependent dioxygenases, including CP4H. The synthesis of 4-hydroxyproline by CP4H is a crucial step for the formation of the collagen triple helix [36], underscoring the involvement of α-KG in collagen biosynthesis.

To validate that the elevated levels of α-KG enhance the chondrogenic differentiation, permeable α-KG (dimethyl (DM)-α-KG) was introduced in chondrogenic induction. This addition resulted in a dose-dependent increase in the formation of AB-positive matrix in 2D-CI (Supplementary Fig. 5A and B), without influencing cell growth (Supplementary Fig. 5B). Furthermore, in the context of 3D-CI with BM-MSCs treated with TGFβ, DM-α-KG increased Safranin O-positive area, reproducing the observed effect of HCA in vitro (Supplementary Fig. 5C).

**Fig. 4:**
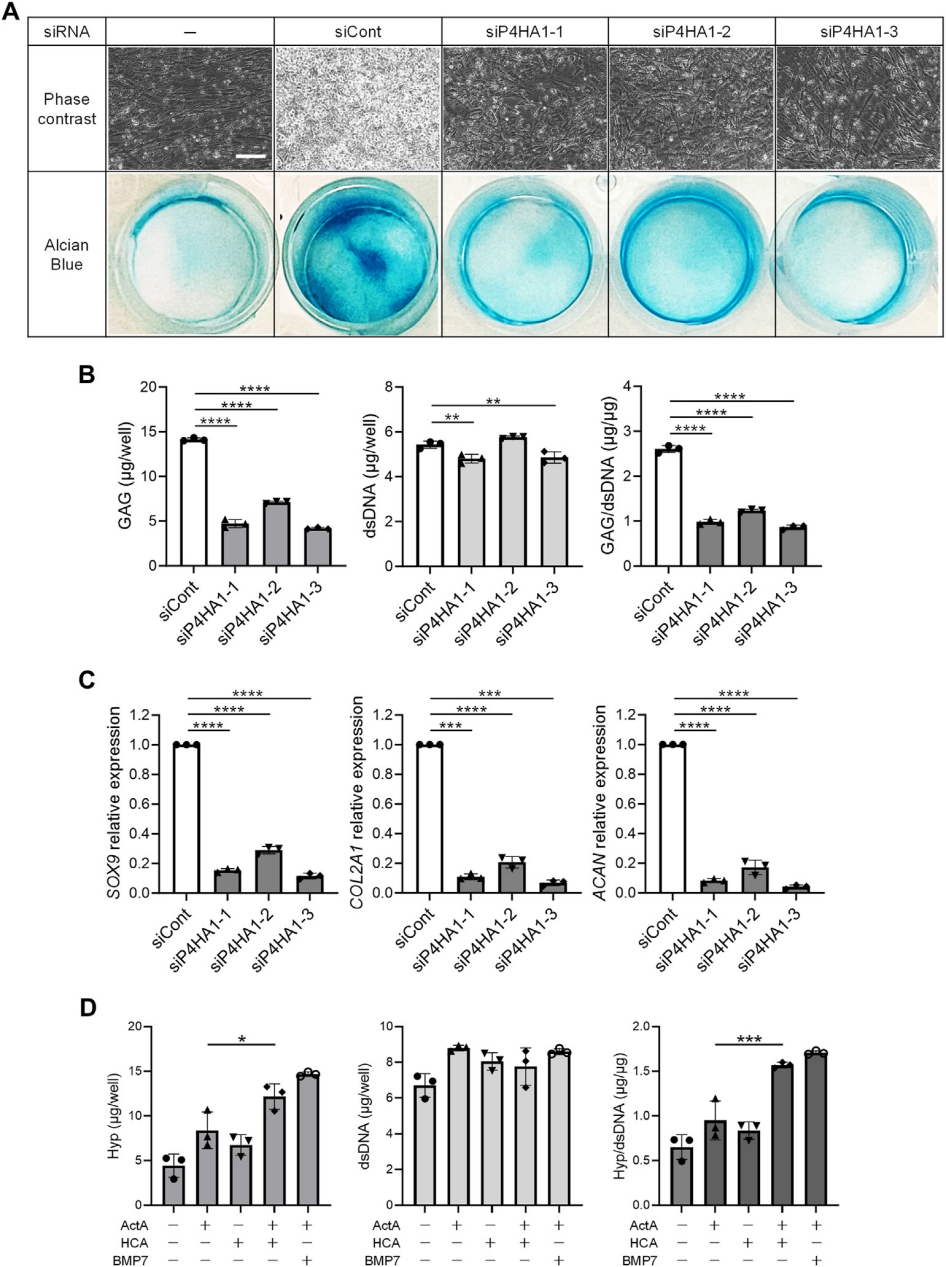
**Hydroxycitric acid (HCA) promoted the chondrogenic differentiation of iPSCs-derived mesenchymal stem cells (iMSCs) in two-dimensional chondrogenic induction (2D-CI) via the alpha-ketoglutarate (α-KG)- collagen prolyl 4-hydroxylase (CP4H) pathway. (A**–**C)** Effect of knockdown of *P4HA1* on 2D-CI. iMSCs were transfected with indicated siRNA at the day before culture initiation and cultured in chondrogenic basal medium with containing Activin A (ActA) (20 ​ng/mL) and HCA (100 ​μM) for 12 days. The assessment was conducted by phase contrast images and AB staining **(A)**, quantification of glycosaminoglycan (GAG) and double stranded DNA (dsDNA) **(B)**, and the measurement of mRNA expression of chondrocyte-related genes **(C)**. The expression level was normalized against samples cultured with siCont. Scale bar ​= ​100 ​μm. **(D)** Effect of HCA on hydroxyproline (Hyp) production. iMSCs were cultured in chondrogenic basal medium containing ActA (20 ​ng/mL), BMP 7 (BMP7) (20 ​ng/mL), and/or HCA (100 ​μM) for 6 days. The assessment was conducted by quantification of Hyp, dsDNA and Hyp/dsDNA ratio. Statistical significance was determined by one-way ANOVA. ∗*P* ​< ​0.05, ∗∗*P* ​< ​0.01, ∗∗∗*P* ​< ​0.001 and ∗∗∗∗*P* ​< ​0.0001). Values are mean ​± ​SD. *n* ​= ​3, independent experiments.

To elucidate the role of CP4H as a downstream factor influenced by α-KG in HCA-induced chondrogenic differentiation, we employed specific siRNAs to suppress the mRNA expression of *P4HA1* gene, which encodes CP4H, during the 2D-CI with ActA and HCA. All three siRNAs targeting *P4HA1*, exhibiting comparable inhibitory effects (Supplementary Fig. 3B), effectively inhibited the formation of AB-positive matrix (Fig. 4A) and the production of GAG per cell (Fig. 4B). Interestingly, the inhibition of *P4HA1* also significantly hampered the expression of chondrogenesis-related genes (Fig. 4C), implying that deficiency of proper triple helix formation transmits a negative signal for chondrogenic differentiation.

Finally, we explored the impact of HCA on hydroxyproline formation. HCA alone failed to increase hydroxyproline level, yet when combined with Activin A, substantial augmentation was observed, which was independent of cell growth (Fig. 4D). These findings imply that the elevated levels of α-KG play a crucial role in facilitating hydroxyproline formation during the chondrogenic induction, particularly in collagen synthesis. Consequently, these results propose that the increase of α-KG is one of mechanisms through which HCA enhances 2D-CI.

### 3.6 HCA promoted the chondrogenic differentiation of iMSCs in 2D-CI by reducing acetylated β-catenin

**Fig. 5:**
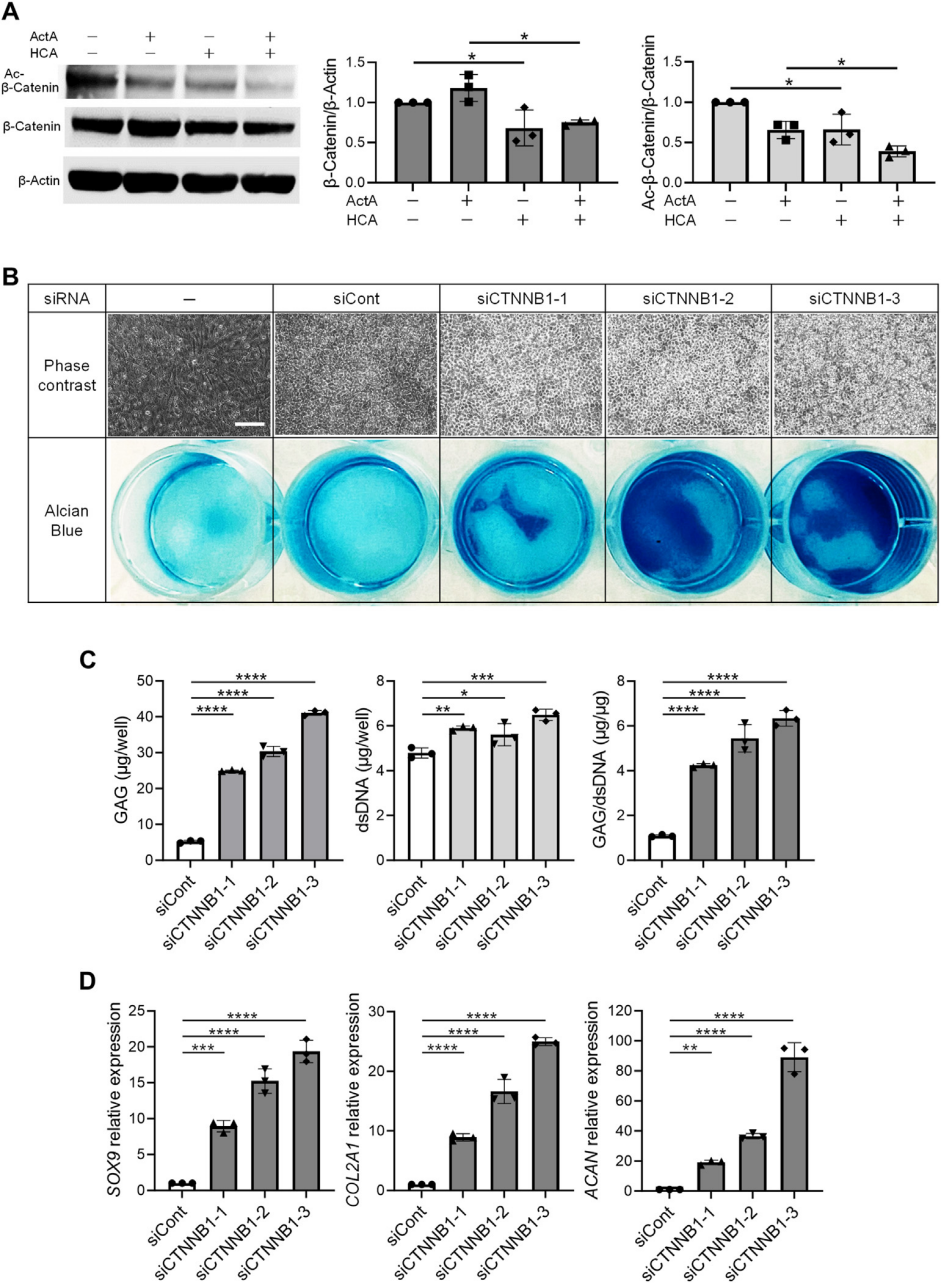
**Hydroxycitric acid (HCA) promoted the chondrogenic differentiation of iPSCs-derived mesenchymal stem cells (iMSCs) in two-dimensional chondrogenic induction (2D-CI) by reducing acetylated β-catenin. (A)** Effect of HCA on β-catenin and Acetylated (Ac)-β-catenin production. iMSCs were cultured in chondrogenic basal medium containing Activin A (ActA) (20 ​ng/mL) and/or HCA (100 ​μM) for 3 days. The levels of Ac-β-catenin and β-catenin protein were evaluated by Western blotting and quantitative densitometry. The relative values of β-catenin/β-actin and Ac-β-catenin/β-catenin in each culture condition were demonstrated as relative in comparison to the ActA (−) and HCA (−) condition. (**B-D)** Effect of the suppression of *CTNNB1* gene on 2D-CI. iMSCs were transfected with indicated siRNA at the day before culture initiation and cultured in chondrogenic basal medium containing ActA (20 ​ng/mL) for 12 days. The assessment was conducted by phase contrast images and AB staining **(B)**, quantification of glycosaminoglycan (GAG) and double stranded DNA (dsDNA) **(C)**, and measurement of mRNA expression of chondrocyte-related genes **(D)**. The expression level was normalized against samples cultured with siCont. Scale bar ​= ​100 ​μm. Statistical significance was determined by one-way ANOVA. ∗*P* ​< ​0.05, ∗∗*P* ​< ​0.01, ∗∗∗*P* ​< ​0.001 and ∗∗∗∗*P* ​< ​0.0001). Values are mean ​± ​SD. *n* ​= ​3, independent experiments.

Cytoplasmic Ac-CoA plays a role in the acetylation of target molecules, which is one of important posttranslational modifications influencing the activity, stability, or intracellular localization of targets [37]. SOX9 is one such molecules affected by the acetylation, which hinders nuclear localization of SOX9 and consequently suppresses the expression of SOX9-regulated genes, including *COL2A1* [38]. Although the expression of SOX9 was induced by the treatment of Activin A, no acetylated SOX9 was detected in cell lysates (Supplementary Fig. 6), suggesting that majority of induced SOX9 was acetylation-free. Therefore, the downregulation of Ac-CoA by HCA was not implicated in the activation of SOX9 in 2D-CI of iMSCs. Another molecule subject to acetylation is β-catenin, and its acetylation contributes to its activity through stabilization [39]. Acetylated β-catenin was detected in cell extracts of iMSCs, and HCA treatment led to a reduction in both acetylated β-catenin and total β-catenin (Fig. 5A), indicating a diminished activity of β-catenin. To investigate the role of β-catenin in 2D-CI, the expression of *CTNNB1* gene encoding β-catenin was inhibited by specific siRNAs. All three siRNAs against *CTNNB1*, with comparable suppressing effects (Supplementary Fig. 3C), significantly enhanced the formation of AB-positive matrix (Fig. 5B) and the production of GAG per cell (Fig. 5C). Furthermore, siRNA treatment up-regulated mRNA expression of chondrogenesis-related genes (Fig. 5D). These results indicate that the inhibition of β-catenin acetylation is one of the roles of HCA in promoting 2D-CI of iMSCs.

Ac-CoA also plays a role of the acetylation of histone, which regulates the gene expression. To evaluate the role of HCA in histone acetylation, the ratio of acetylated H3 to total H3 was analyzed in chondrogenic induction (Supplementary Fig. 7). No clear reduction of acetylated H3 fraction was observed by HCA treatment, suggesting that the histone acetylation is not involved in the effect of HCA for the chondrogenic induction in our experimental system.

### 3.7 HCA promoted the chondrogenic differentiation of iMSCs in 2D-CI by enhancing mitochondrial activity

**Fig. 6:**
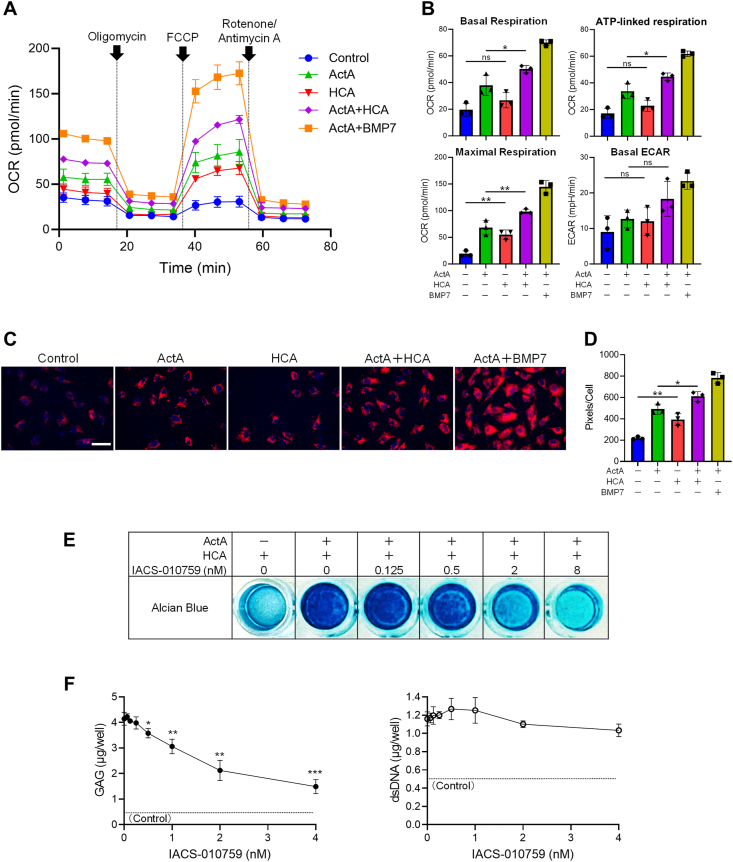
**Hydroxycitric acid (HCA) promoted the chondrogenic differentiation of iPSCs-derived mesenchymal stem cells (iMSCs) in two-dimensional chondrogenic induction (2D-CI) by enhancing mitochondrial activity. (A**–**B)** The effect of HCA on OCR in 2D-CI. iMSCs were cultured in chondrogenic basal medium containing Activin A (ActA) (20 ​ng/mL), BMP 7 (BMP7) (20 ​ng/mL), and/or HCA (100 ​μM) for 6 days, and the profile of OCR **(A)** and the value of each parameter **(B)** was analyzed using a Seahorse flux analyzer. (***C*-D)** The effect of HCA on the mitochondrial membrane potential. iMSCs were cultured in chondrogenic basal medium containing ActA (20 ​ng/mL), BMP7 (20 ​ng/mL), and/or HCA (100 ​μM) for 6 days and then stained by TMRE (red) and Hoechst33342 (blue) **(C)**. The intensity of TMRE staining was quantified by signal per cell **(D)**. **(E**–**F)** The effect of mitochondrial inhibitor, IACS-010759, on 2D-CI. iMSCs were cultured in chondrogenic basal medium containing ActA (20 ​ng/mL) and/or HCA (100 ​μM) with indicated concentration of IACS-010759 for 12 days. The assessment was conducted by AB staining **(E)** and quantification of glycosaminoglycan (GAG) and double stranded DNA (dsDNA) **(F)**. Statistical significance was determined using one-way ANOVA **(B** and **D)** and Student's *t*-test compared to samples treated without IACS-010759 **(F)**. Values are mean ​± ​SD. ∗*P* ​< ​0.05, ∗∗*P* ​< ​0.01 and ∗∗∗*P* ​< ​0.001. *n* ​= ​3, independent experiments.

In our recent findings, we highlighted the significance of mitochondrial activity in the chondrogenic differentiation of iMSCs [40]. To explore the impact of HCA on the mitochondrial activity of iMSCs during 2D-CI, we analyzed OCR profiles in cells cultured with various factors (Fig. 6A) and assessed each of basal respiration, ATP-linked respiration, maximal respiration and basal extracellular acidification rate (ECAR) (Fig. 6B). iMSCs cultured with ActA and BMP7 exhibited upregulation in all three parameters for OXPHOS, while basal ECAR did not show significant change. While treatments with ActA or HCA alone did not lead to an increase of ATP-linked respiration, a notable elevation was observed by the combined treatment. Regarding maximal respiration, both ActA and HCA individually showed a significant increase, which was further augmented by their combination. These data suggested that HCA itself possesses an activity to enhance mitochondrial function. Further analysis in living cells, using TMRE incorporation (Fig. 6C) and subsequent quantification (Fig. 6D), revealed that HCA itself induced the mitochondrial activity and synergistically enhanced the effect of ActA. Treatment with IACS-017059, an inhibitor of complex I of oxidative phosphorylation (OXPHOS) [41] hindered the formation of AB-positive matrix (Fig. 6E) and the production of GAG per cell (Fig. 6F) in iMSCs treated with ActA and HCA. Importantly, these results were observed at the concentration of IACS-017059 that exhibited no impact on cell growth, suggesting that the enhancement of mitochondrial activity is one of mechanisms through which HCA promotes chondrogenesis.

### 3.8 β-Catenin inhibited the chondrogenic differentiation of iMSCs by suppressing the activity of OXPHOS

**Fig. 7:**
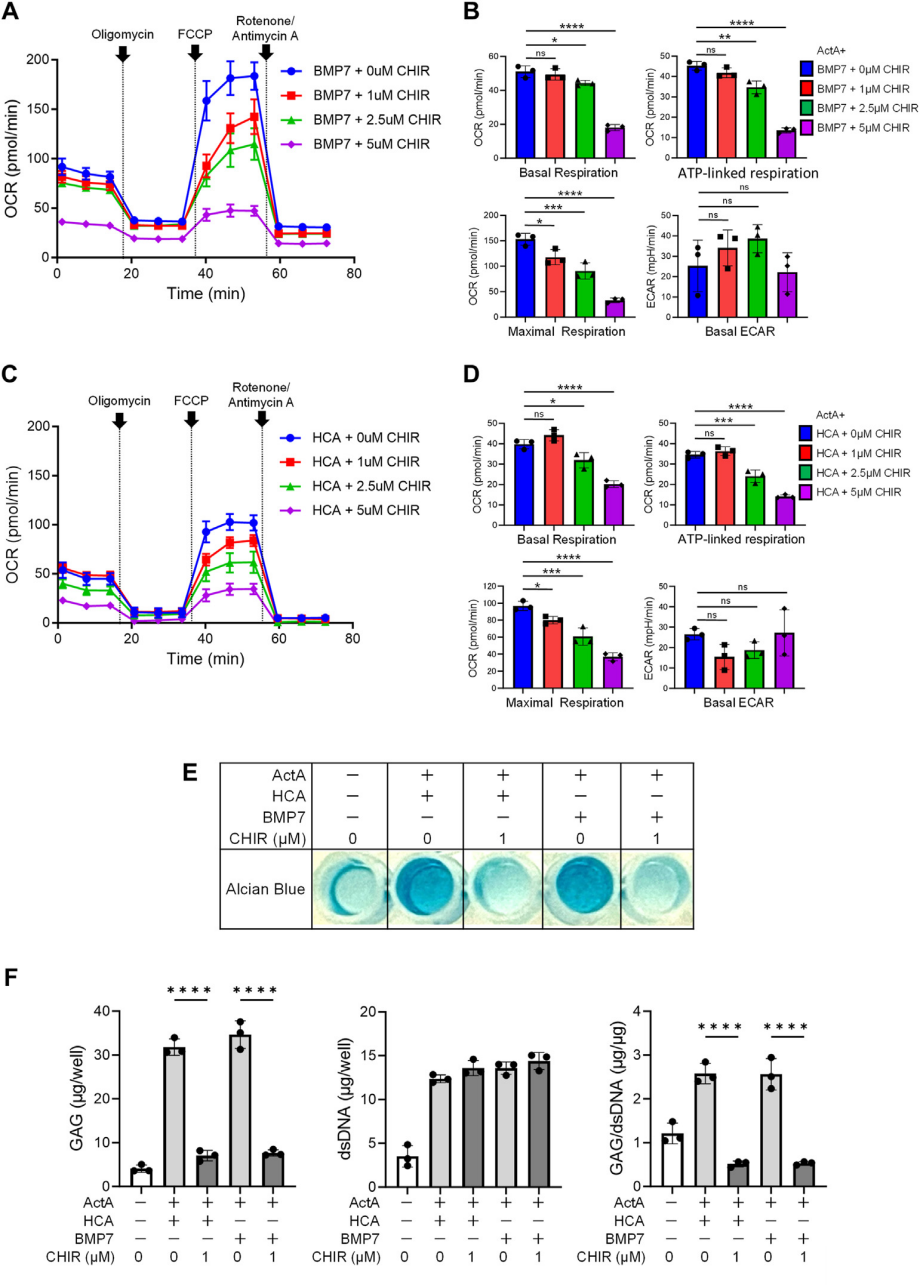
**Activation of β-catenin suppressed OXPHOS and chondrogenic differentiation of iPSCs-derived mesenchymal stem cells (iMSCs) in two-dimensional chondrogenic induction (2D-CI). (A**–**D)** The effect of CHIR-99021 (CHIR) on OCR in 2D-CI. iMSCs were cultured in chondrogenic basal medium containing Activin A (ActA) (20 ​ng/mL), BMP 7 (BMP7) (20 ​ng/mL) and indicated concentration of CHIR **(A**–**B)**, or ActA (20 ​ng/mL), hydroxycitric acid (HCA) (100 ​μM) and indicated concentration of CHIR **(*C***–**D)** for 6 days, and the profile of OCR **(A** and **C)** and the value of each parameter **(B** and **D)** was analyzed using a Seahorse flux analyzer. **(E**–**F)** The effect of CHIR on chondrogenic differentiation in 2D-CI. iMSCs were cultured in chondrogenic basal medium containing ActA (20 ​ng/mL), BMP7 (20 ​ng/mL), and/or HCA (100 ​μM) with indicated concentration of CHIR for 12 days. The assessment was conducted by AB staining **(E)** and quantification of glycosaminoglycan (GAG) and double stranded DNA (dsDNA) **(F)**. Statistical significance was determined using one-way ANOVA. ∗*P* ​< ​0.05, ∗∗*P* ​< ​0.01, ∗∗∗*P* ​< ​0.001 and ∗∗∗∗*P* ​< ​0.0001. Values are mean ​± ​SD. *n* ​= ​3, independent experiments.

The inhibitory effect of Wnt/β-catenin signal for chondrogenesis has been documented [42], and the inhibitor for Wnt/β-catenin signal has been employed in clinical trials for OA [43]. However, the precise mechanisms by which Wnt/β-catenin signal impedes chondrogenic differentiation remain elusive. In light of our findings demonstrating that HCA reduces β-catenin levels and enhances the mitochondrial activity, we explored the interplay between Wnt/β-catenin signaling and mitochondrial activity using CHIR-99021, a GSK-3β inhibitor [44]. The phosphorylation of β-catenin by GSK-3β leads to its destabilization, and CHIR-99021 protects β-catenin from the degradation, and thereby amplifying the Wnt/β-catenin signal [44]. iMSCs cultured in 2D-CI condition for five days were treated with CHIR-99021 for 24 h, and OCR of them were subsequently calculated. The heightened OCR by the treatment with ActA and BMP7 were markedly downregulated in a dose-dependent manner by CHIR-99021 (Fig. 7A and B). Similar outcomes were observed in 2D-CI using ActA and HCA, where each parameter of OCR was dose-dependently reduced by CHIR-99021 (Fig. 7C and D). Importantly, the 24 h treatment with CHIR-99021 did not exhibit cytocidal effects for iMSCs, suggesting that CHIR-99021 directly impacted mitochondrial activity.

Long-term treatment of 1 μM of CHIR-99021 exhibited no discernible effects for cell growth. However, it demonstrated the inhibition of AB-positive matrix formation (Fig. 7E) and GAG production (Fig. 7F). To further substantiate the involvement of Wnt/β-catenin signal in OCR, iMSCs subjected to chondrogenic induction were treated with siRNA targeting *CTNNB1* for 6 days and OCR was subsequently analyzed (Supplementary Fig. 8A). The administration of siRNA targeting *CTNNB1* alone resulted in the upregulation of basal respiration and maximal respiration. When combined with Activin A, this combination effectively upregulated all three OCR parameters (Supplementary Fig. 8B). Integrating these findings with the data of CHIR-99021 treatment, it is evident that the inhibition of mitochondrial activity represents at least one of mechanisms underlying the suppressive effects of Wnt/β-catenin signal on chondrogenesis.

### 3.9 Orally administered HCA reconstructed articular cartilage by modulating metabolites in knee joints of mice

**Fig. 8:**
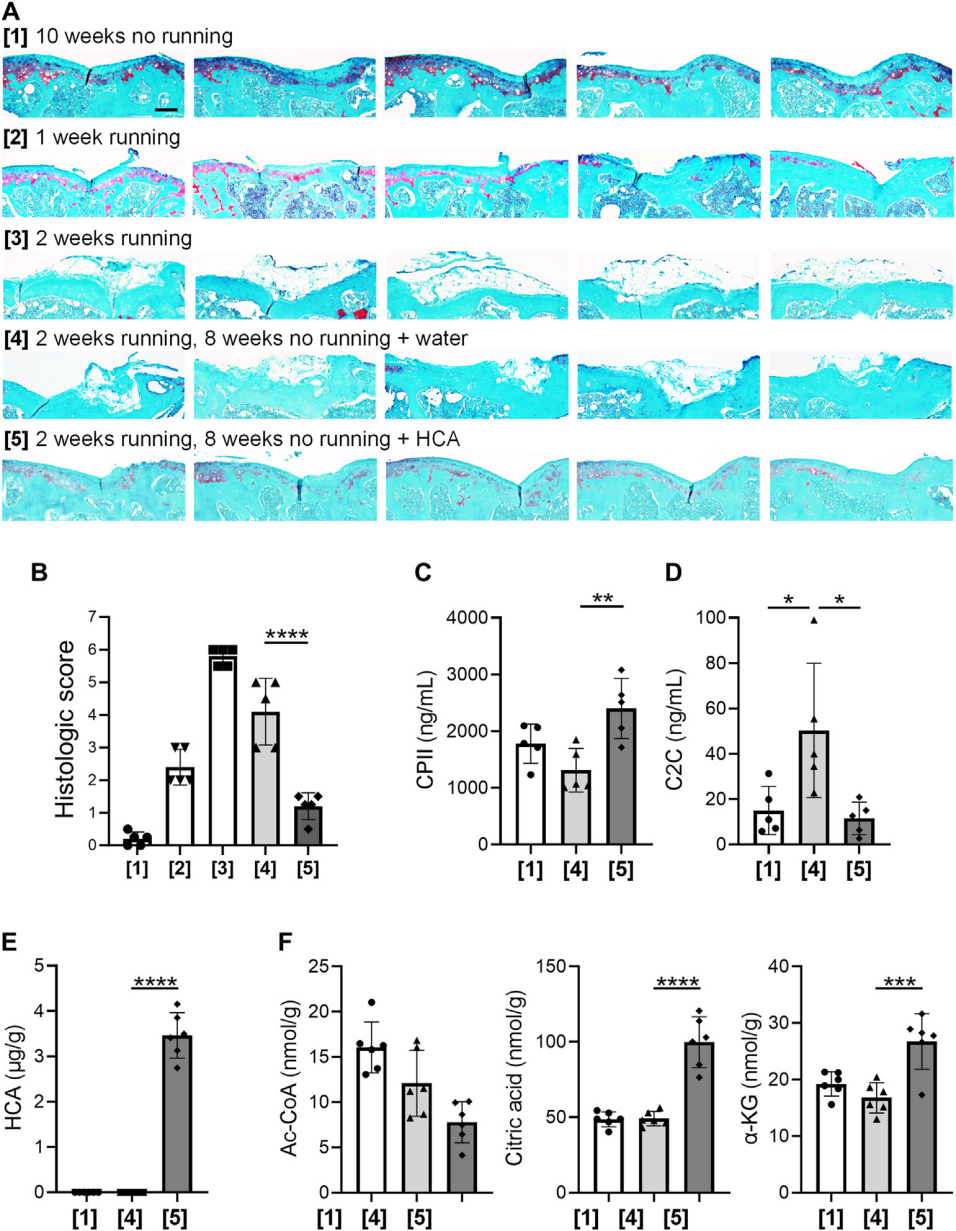
**Orally administered hydroxycitric acid (HCA) reconstructed damaged articular cartilage by modulating metabolites in knee joints of mice**. **(A)** Histological evaluation of articular cartilage. Mice from each group ([[1], [2], [3], [4], [5]]) were sacrificed at the end of each schedule and sections of articular cartilage from the medial condyles of femurs were stained by Safranin O. Scale bar indicates 100 ​μm. **(B)** Evaluation of the quality of articular cartilage by the OARSI grading system. **(*C***–**D)** Evaluation of cartilage metabolic markers in blood. The levels of *c*-propeptide of type II procollagen (CPII) **(C)** and collagen Type II cleavage (C2C) **(D)** was quantified in blood collected at the time of sacrifice. *n* ​= ​5, independent experiments. **(E**–**F)** Quantitative determination of HCA **(E)** and acetyl coenzyme A (Ac-CoA), citric acid, and alpha-ketoglutarate (α-KG) **(F)** in extracts of joint tissues. *n* ​= ​6, independent experiments. Statistical significance was determined using one-way ANOVA. ∗*P* ​< ​0.05, ∗∗*P* ​< ​0.01, ∗∗∗*P* ​< ​0.001 and ∗∗∗∗*P* ​< ​0.0001. Values are mean ​± ​SD.

In vitro experiments demonstrated that HCA promoted chondrogenic differentiation of MSC through multiple mechanisms, modifying the metabolic cascade. While HCA alone exhibited minimal effect on chondrogenic differentiation, its combination with TGFβ signal induced by inflammation may exert its potential to augment the intrinsic regenerative properties of injured cartilage tissue. To explore the impact of HCA in an in vivo setting, we established a mouse model developing OA through exercise. To correctly evaluate the effect of running and HCA treatment, sections at the same level from each sample were selected based on the outline and growth plate morphology (Supplementary Fig. 9). Histological examination of distal femoral articular cartilage tissues showed that daily forced running on an incline induced cartilage degeneration as early as one week (Fig. 8A, [2]), progressing complete destruction of articular surface after two weeks (Fig. 8A, [3]). In contrast, cartilage structures were maintained in mice without forced running for 10 weeks (Fig. 8A, [1]), affirming the efficiency of our model in inducing cartilage degeneration and the suitability for evaluating the therapeutic interventions. The degeneration process was quantitatively demonstrated by the score using the grading system (Fig. 8B). No significant difference of body weight change profiles was observed during the experiments between mice with and without HCA treatment (Supplementary Fig. 10).

Mice given only water for hydration showed elimination of cartilage tissues at 8 weeks after two weeks of running (Fig. 8A [4], and Fig. 8B), whereas mice orally administrated with HCA demonstrated cartilaginous tissues in area where cartilage tissues were expected to have been depleted due to running (Fig. 8A, [5]). Morphometric score reached to a level comparable to that observed after one week of running (Fig. 8B).

The impact of running and HCA treatment were further assessed by the serum levels of biomarkers for cartilage metabolism. *C*-propeptide of type II collagen (CPII) is a carboxypropeptide that is cleaved from type II procollagen after newly synthesized procollagens are released into the cartilage [45]. As such, it serves as a marker of cartilage synthesis. Collagen Type II cleavage (C2C), a marker of cartilage degradation, is a neopeptide produced by collagenase cleavage (MMP-1, MMP-8, MMP-13) of type II collagen [46]. The running exercise for two weeks showed minimal impact of CPII levels, whereas the oral administration of HCA for 8 weeks significantly increased the level of CPII (Fig. 8C). The serum level of C2C showed a significant increase after running period and remained elevated at 8 weeks post-running, suggesting the continuous progression of OA changes. This increase was significantly reduced by the administration of HCA to levels comparable to those observed in non-running mice (Fig. 8D). Given that cartilage degeneration had already occurred before HCA administration, the bidirectional effect of HCA played a crucial role in contributing to the reconstruction process.

To validate that the observed therapeutic effects were a result of administrated HCA, metabolites in extracts of joint tissues were analyzed. At first, the presence of HCA in joint tissues was confirmed by liquid chromatography-mass spectrometry (LC-MS/MS) (Fig. 8E). Regarding metabolites, consistent with in vitro data (Fig. 3D, E and 3F), the amount of Ac-CoA was reduced, while the levels of citrate and α-KG were upregulated in HCA-treated mice (Fig. 8F). These findings suggest that the effect of HCA in inducing cartilage tissue is attributed to the modification of metabolites through the inhibition of ACLY.

## 4 Discussion

In this study, we demonstrated that a chemical capable of enhancing the chondrogenic potential of MSCs with TGFβ, by modulating the metabolomic pathway in vitro, also promotes the reconstruction of damaged articular cartilage in a similar manner. OA stands as one of the most prevalent chronic diseases globally and is a leading cause of disability. In Japan, two cohort studies revealed that over 60 ​% of residents aged 60 years and older experienced radiologically-defined painful knee OA [47,48]. Given the high prevalence and gradual progression of this disease, the oral administration of symptomatic slow-acting drugs emerges as an ideal treatment choice. While certain randomized clinical trials investigating chondroitin sulfate or glucosamine sulfate reported favorable outcomes [49,50], others contradicted these findings [51,52], and a meta-analysis of reported RCT data failed to confirm the benefit of uptake [52]. In this context, our study demonstrated the possibility of HCA as one of orally administered therapeutic agents for OA.

HCA is a major constituent of *Garcinia*, which is an extract from fruits of *Garcinia cambogia* and has been widely employed as a weight-loss supplement [53]. The observed effect is credited to the anti-obesity activity of HCA, which inhibits the activity of ACLY [54]. ACLY plays a pivotal role in linking glucose metabolism to lipid metabolism by converting mitochondria-derived citrate into oxaloacetate and Ac-CoA [55]. Ac-CoA, serving as a critical precursor for fatty acid synthase, is integral to the syntheses of fatty acid, cholesterol, and triglyceride syntheses [56], and thus HCA suppresses de novo lipogenesis. Our study unveiled a novel function of HCA, demonstrating its chondroanabolic effect and providing evidence for its application in OA as a therapeutic chemical.

We have elucidated multiple mechanisms through which HCA promotes chondrogenesis. Inhibition of ACLY by HCA results in the modification of two crucial metabolites, namely Ac-CoA and citric acid. Apart from its role in lipogenesis, Ac-CoA serves as a primary donor of acetyl moieties for various acceptor molecules, including amino groups on proteins [35]. Acetylation profoundly influences proteins in terms of their stability, localization, and function [57]. The acetylation of β-catenin at lysine 49 plays a crucial role in inhibiting its phosphorylation and the subsequent ubiquitination-associated proteolysis. This process ultimately leads to an increase in the levels of β-catenin protein [58]. Our study unveiled that HCA diminished the acetylation in β-catenin by reducing Ac-CoA production, consequently causing a decline in its protein expression. Given the abnormal upregulation of Wnt/β-catenin signaling pathway in OA, targeting its inhibition emerges as a rational approach for disease-modifying OA drugs [59]. However, the role of Wnt/β-catenin pathway in chondrogenesis and cartilage regeneration varies across differentiation stages, with specific molecular mechanisms remaining undisclosed. A recent study highlighted that a Wnt/β-catenin signal antagonist induced MSC chondrogenesis by enhancing adherent junction-β-catenin interaction [60]. The present study contributes novel insights by revealing that β-catenin suppresses chondrogenic differentiation of MSC through the inhibition of chondrogenesis-related OXPHOS activation.

It is established that the mitochondrial activity is dispensable for the development of growth plate cartilage [61]. However, in instances of heterotopic cartilage formation, resembling the regeneration process, we have discovered the indispensable role of OXPHOS activation [40]. The regeneration process is likely associated with protein synthesis, requiring ATP for new protein production and proper protein folding. To date, ACLY has been acknowledged as an OXPHOS activator in skeletal muscles [55] or cancer cells [62] by diverse mechanisms. In contrast, our findings indicate that ACLY exerts a negative regulatory effect on OXPHOS by stabilizing the β-catenin protein, implying a cell type-dependent function of ACLY. Notably, higher expression of ACLY was detected in OA chondrocytes compared to healthy chondrocytes [38]. In light of our data, this elevated ACLY expression may facilitate the degeneration process by stabilizing the β-catenin protein. The existence of the ACLY-β-catenin axis in OA chondrocytes poses a critical question for rationalization of the *anti*-Wnt/β-catenin therapy for OA. Addressing this issue is pivotal for understanding the potential impact of targeting this axis in developing therapeutic strategies for OA.

The accumulation of citrate induced by HCA leads to an increase of α-KG, a key metabolite in the tricarboxylic acid cycle and OXPHOS activation. Additionally, α-KG serves as an important rate-limiting cofactor of prolyl 4-hydroxylase, including collagen prolyl 4-hydroxylase [63]. The administration of α-KG enhances the stability of collagen helices through increased proline hydroxylation, resulting in a higher abundance of type II collagen-positive cartilage [64]. We also found that chondrogenesis with exogeneous α-KG increased the formation of AB-positive matrix (Supplementary Fig. 5). It is noteworthy and conceivable that the diminished production of extracellular matrix (ECM) by inhibiting CP4H activity disrupts the expression of early chondrogenesis-related genes, highlighting a crosstalk between ECM and transcription factors. The ECM not only serves as a product but also regulates chondrogenesis, providing a cell type-specific microenvironment. For instance, *N*-cadherin and *N*-CAM, molecules involved in cell-cell interaction and adhesion, contribute to cellular condensation and subsequent chondrogenesis [65]. Moreover, integrins transmit signals from the ECM to intracellular effectors, and their knockout results in chondrodysplasia-like phenotype [66,67]. Hence, the ECM plays a dual role as both a product and a regulator in the process of chondrogenesis.

In this study, the in vivo results demonstrate the efficacy of HCA in producing cartilaginous tissues. Various methods exist for creating OA models in mice, with most being post-traumatic models involving surgical interventions like ACL transection combined with medial meniscus resection [68]. Clinical data indicates that such injuries can lead to OA if not properly treated [69]. However, many OA patients do not experience definite trauma episodes, and repetitive mechanical stress is considered to be a major factor [7]. Our exercise-induced model might be more appropriate for evaluating therapeutic drug for OA. Histological assessments demonstrate HCA's ability to produce cartilaginous tissues after the destruction of articular cartilages structures. Metabolites profiles in joint tissues align with those observed in vitro, and systemic evaluation shows an increase in an anabolic marker. These findings strongly suggested that HCA exerted its chondroanabolic action in vivo, indicating potential therapeutic application for the symptomatic patients.

Although we did not present evidence for the existence of TGFβ signal in degenerated joints, administrated HCA existed in knee joints and metabolic profiles align with in vitro data. These data suggest that HCA likely promotes chondrogenesis through mechanisms similar to those demonstrated in vitro, potentially involving local TGFβ signals. We currently lack data on which types of cells in knee joints respond to HCA and contribute to tissue reconstruction. The disruption of tidemark, separating calcified and non-calcified chondrocytes, could facilitate the invasion of bone marrow stromal cells, possibly including MSCs, in surface areas [1]. Additionally, synovium-derived MSCs may migrate to joint surface by inflammatory signals [1,18]. It is also plausible that cells with stem cell properties in articular cartilage tissue play a role in reconstruction [1,18]. Performing similar experiments using mice with some lineage-tracing systems could offer intriguing insights into these processes.

OA is a condition that typically begins in middle age and progress gradually over time. In a review article analyzing 16 randomized placebo-control studies, no differences were found in humans in terms of side effects or adverse events between groups treated with Garcinia cambogia and the placebo groups [70]. Given the chondroanabolic effect of orally administered HCA at a dose equivalent to the approved human daily dosage, the combination therapy with matrix-related slow-acting drugs like chondroitin sulfate or glucosamine sulfate presents an intriguing avenue for assessing the therapeutic potential of HCA.

This study has several limitations. As mentioned above, we did not demonstrate the presence or contribution of stem cells in the reconstructed articular cartilage in vivo, although MSCs were used in the in vitro study. In addition, the presence and source of TGFβ signaling during reconstruction was not investigated. As only a single dose of HCA was used in the in vivo experiments, we were not able to evaluate the dose-dependent effects, which are the main evidence for the efficacy of HCA.

## Author contributions

Y.M. conducted most of experiments, data extraction, and analysis. L.S. performed OCR analyses. M.M., S.N., T.K., and M.F. provided assistance with experiments. K.T. contributed to the study design. J.T. supervised the project and aided in manuscript preparation. Y.J. conceived and designed the study, analyzed the data, and drafted the manuscript. All authors read and approved the final manuscript.

## Declaration of competing interest

Y.M. and K.T. are employees of Kobayashi Pharmaceutical Co., Ltd. but and declare no non-financial competing interests. All other authors declare no financial or non-financial competing interests.
