# Supplementary material for: Hydroxycitric acid reconstructs damaged articular cartilages by modifying the metabolic cascade in chondrogenic cells
Source: Osteoarthr Cartil Open. 2024 Dec 24;7(1):100564. doi: 10.1016/j.ocarto.2024.100564 (PMC11743121; doi:10.1016/j.ocarto.2024.100564)
Supplement: Multimedia component 1 [file mmc1.pdf]

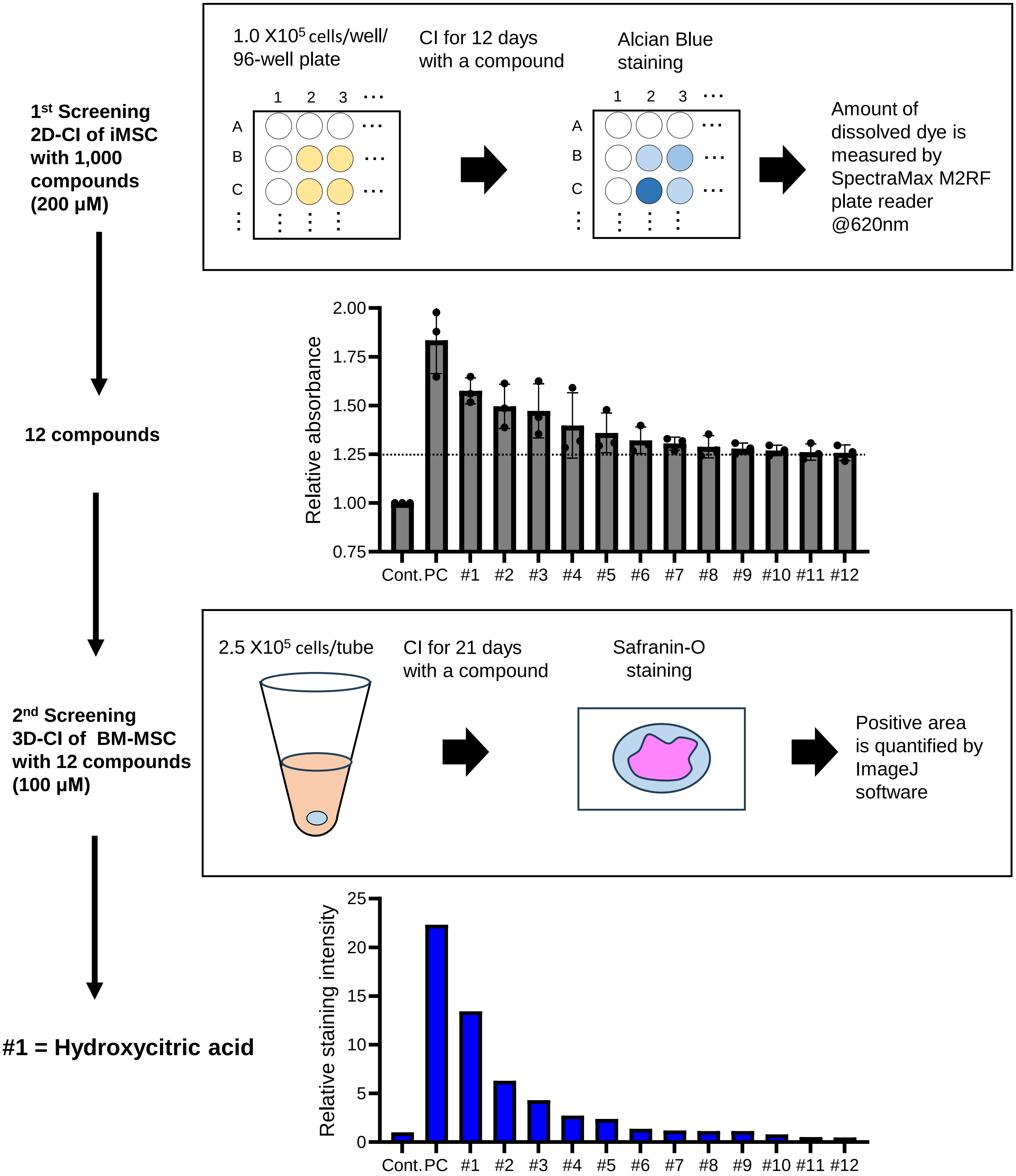

Supplementary Figure 1. Schematic demonstration of the screening process. The first screening was two-dimensional chondrogenic induction (2D-CI) of iPSCs-derived MSCs (iMSCs) and evaluated by the formation of Alcian Blue positive matrix. Out of 1,000 chemical compounds (200 µM), 12 compounds exhibited a relative absorbance more than 1.25 times higher than the control, which was treated with Activin A alone. The second screening was three-dimensional chondrogenic induction (3D-CI) of bone marrow-derived MSCs (BM-MSC) using these 12 compounds (100 µM) and transforming growth factor-beta 3 (TGFβ3) (5 ng/mL) and evaluated by Safranin O positive area in histological sections. As results, hydroxycitric acid (HCA), identified as #1, showed the strongest chondrocyte differentiation-inducing activity in both the first and second screening.

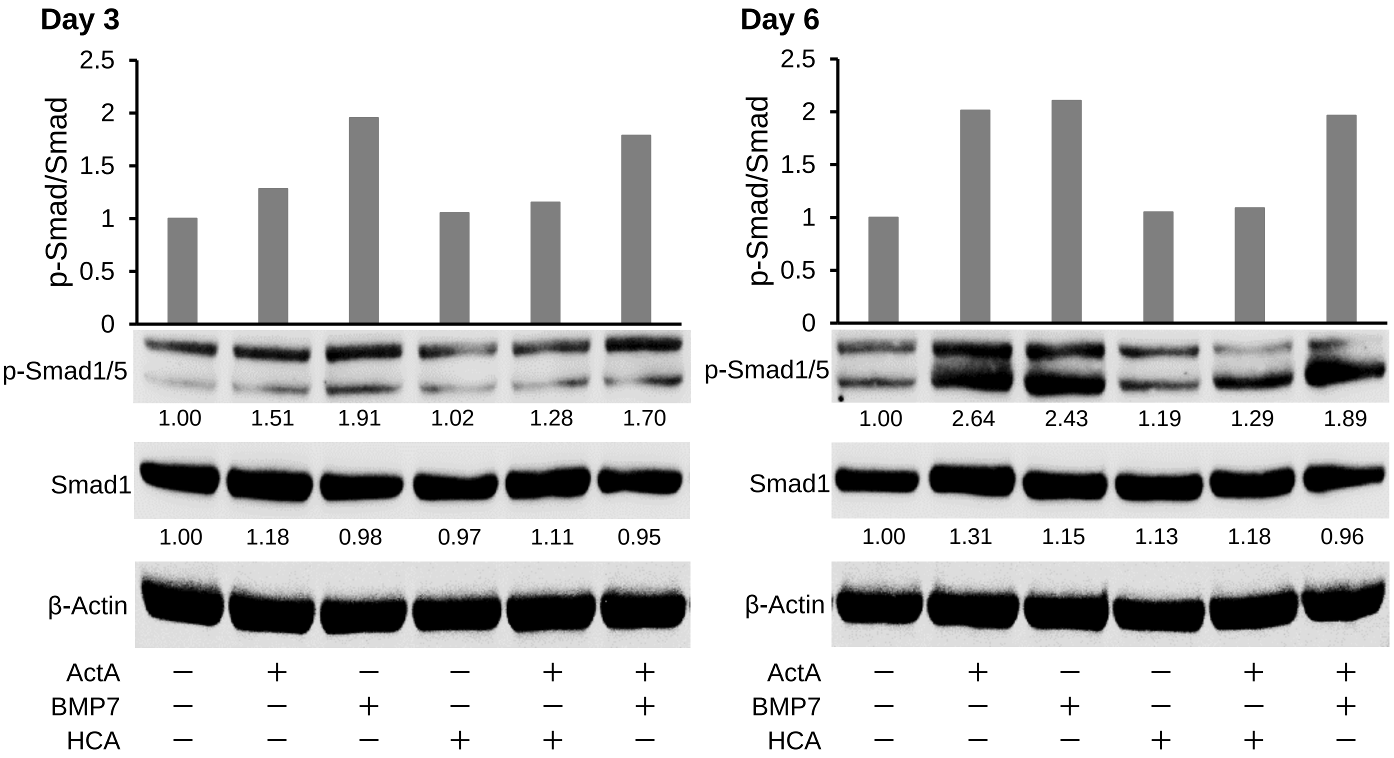

Supplementary Figure 2. Effect of hydroxycitric acid (HCA) on bone morphogenetic protein (BMP) signaling in iPSCs-derived MSCs (iMSCs) on two-dimensional chondrogenic induction (2D-CI). iMSCs were cultured in chondrogenic basal medium containing Activin A (ActA) (20 ng/mL), BMP7 (20ng/mL), and/or HCA (100 µM) for 3 or 6 days, followed by protein extraction and analyzed by Western blotting. Intensity of each protein signal was quantified using densitometry and normalized against samples treated solely with basal medium. The ratio of p-Smad1/5 to Smad1 of each sample was normalized against samples treated solely with basal medium.

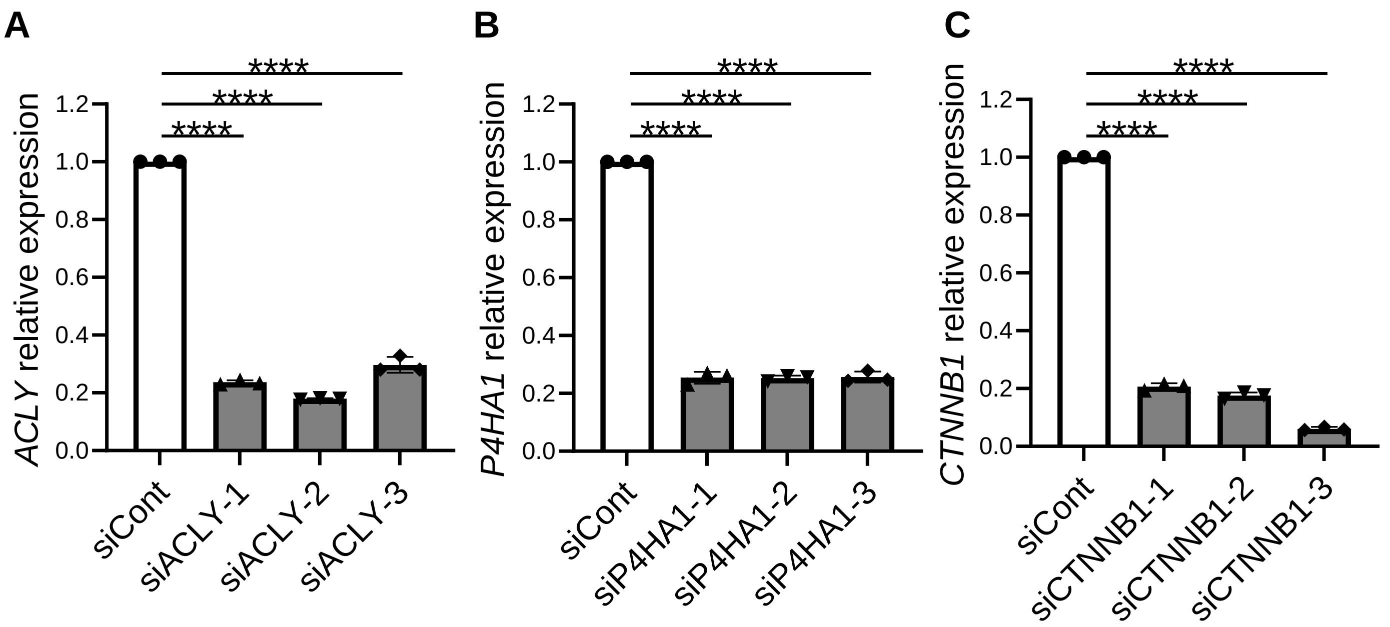

Supplementary Figure 3. Efficacy of siRNAs for target genes in iPSCs-derived MSCs (iMSCs) on two-dimensional chondrogenic induction (2D-CI). iMSCs were transfected with indicated siRNAs for *ACLY* **(A)**, *P4HA1* **(B)**, or *CTNNB1* **(C)** gene at the day before culture initiation. Subsequently, they were subjected to 2D-CI with Activin A (20 ng/mL) (**A** and **C**), or Activin A and hydroxycitric acid (HCA) (100 µM) (**B**). mRNA was collected on day 12, and the expression of each gene was quantified by RT-qPCR. The expression level of each gene was normalized against samples treated with siRNA negative control (siCont). Statistical significance was determined using one-way ANOVA. ****$P < 0.0001$. Values are mean ± SD. *n*=3, independent experiments.

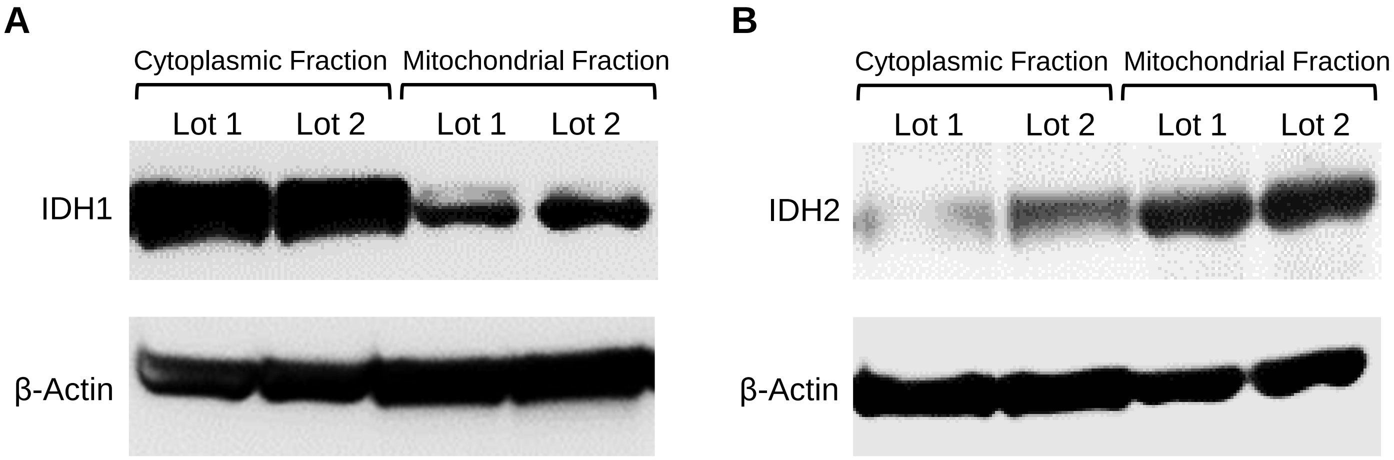

Supplementary Figure 4. Characteristics of cytoplasmic and mitochondrial fractions. iPSCs-derived MSCs **(**iMSCs) were cultured solely in chondrogenic basal medium for 6 days. Subsequently, cytoplasmic and mitochondrial fractions were prepared and analyzed using Western blotting. Isocitrate dehydrogenase 1 (IDH1) **(A)** and IDH2 **(B)** were used as a marker for cytoplasmic or mitochondrial protein, respectively. Two individual experiments (Lot 1 and Lot 2) were performed.

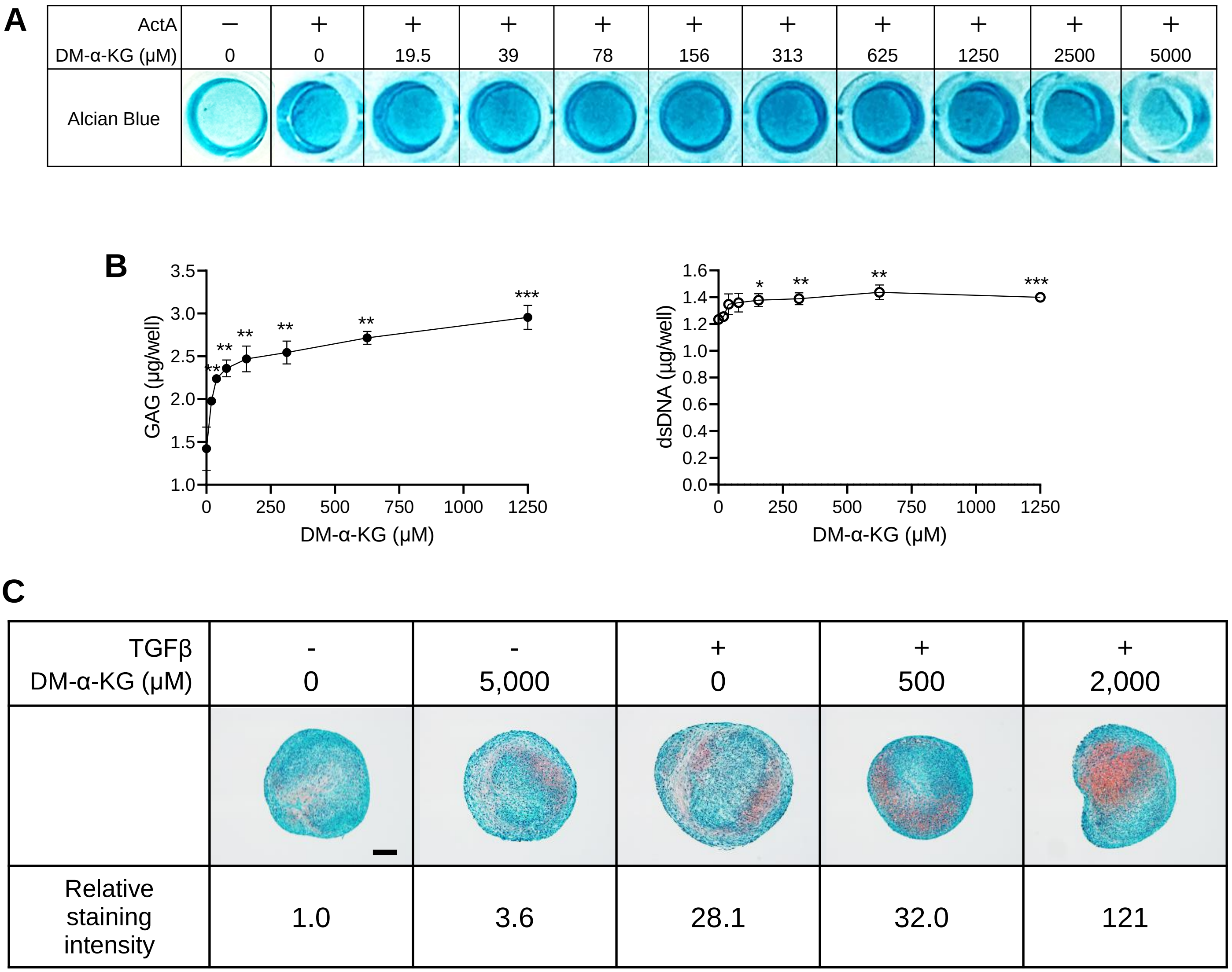

Supplementary Figure 5. Effect of exogenous alpha-ketoglutarate (α-KG) on chondrogenic differentiation. (A-B) Two-dimensional chondrogenic induction (2D-CI) of iPSCs-derived MSCs (iMSCs). iMSCs were cultured in chondrogenic basal medium containing Activin A (ActA) (20 ng/mL) and dimethyl-α-KG (DM-α-KG) at indicated concentration for 12 days. The assessment was conducted by Alcian Blue staining (**A**) and quantification of glycosaminoglycan (GAG) and double stranded DNA (dsDNA) production (**B**). **(C)** Three-dimensional chondrogenic induction (3D-CI) of bone marrow-derived MSCs (BM-MSCs). BM-MSCs were cultured in chondrogenic basal medium containing transforming growth factor-beta 3 (TGFβ3) (5 ng/mL) and DM-α-KG at indicated concentrations in 3D condition for 21 days. Pellets were stained with Safranin O. Relative staining intensity of each section was demonstrated as the ratio of Safranin O positive area in each sample compared to that in samples cultured solely in basal medium. Scale bar indicates 200 μm. Statistical analysis was performed by Student's *t* test compared to samples treated solely with Activin A (**B**). **P* < 0.05, ***P* < 0.01, and ****P* < 0.001. Values are mean ± SD. *n*=3, independent experiments.

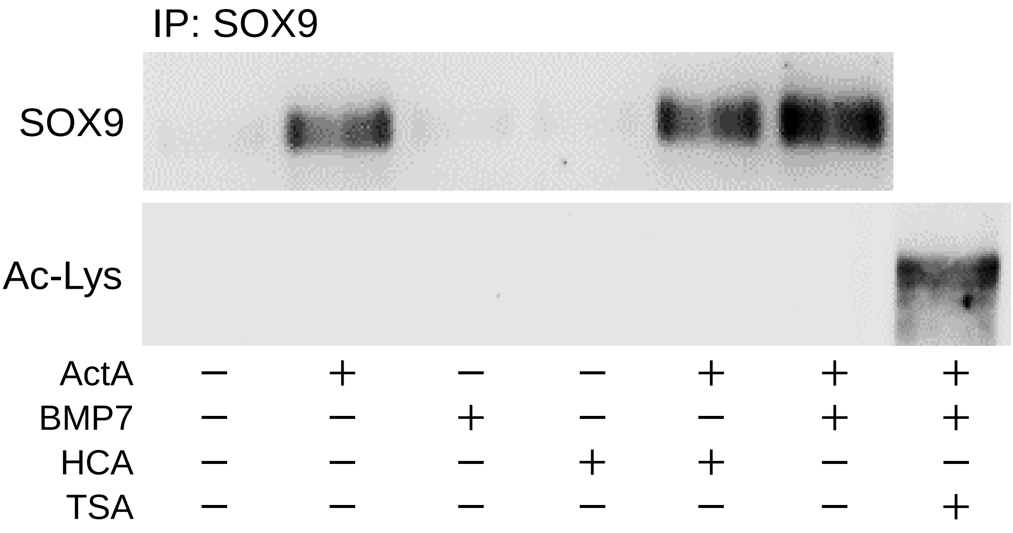

Supplementary Figure 6. Expression of SOX9 and acetylated SOX9 in iPSCs-derived MSCs (iMSCs) on two-dimensional chondrogenic induction (2D-CI). iMSCs were cultured in chondrogenic basal medium containing Activin A (ActA) (20 ng/mL), bone morphogenetic protein 7 (BMP7) (20ng/mL), and/or hydroxycitric acid (HCA) (100 µM) for 3 days, followed by protein extraction and immunoprecipitation with anti-SOX9 antibody. The immunoprecipitated proteins were then analyzed by Western blotting using anti-SOX9 or anti-Ac-Lys antibody. As a positive control, acetylation of lysine residue was induced by the treatment with Trichostatin (TSA).

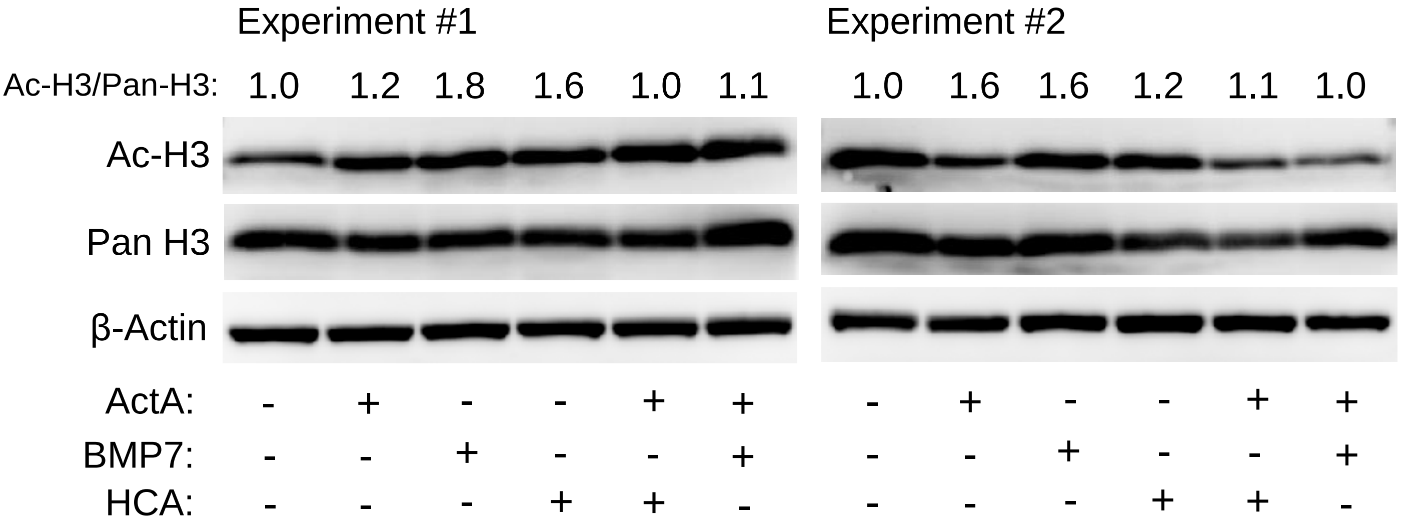

Supplementary Figure 7. Expression of total and acetylated histone H3 (Ac-H3) in iPSCs-derived MSCs (iMSCs) on two-dimensional chondrogenic induction (2D-CI). iMSCs were cultured in chondrogenic basal medium containing Activin A (ActA) (20 ng/mL), bone morphogenetic protein 7 (BMP7) (20ng/mL), and/or hydroxycitric acid (HCA) (100 μM) for 3 days, followed by protein extraction and Western blot analyses using anti-Ac H3, anti-Pan H3, or anti-β-actin antibody. The ratio of Ac-H3 to total H3 was calculated by densitometer.

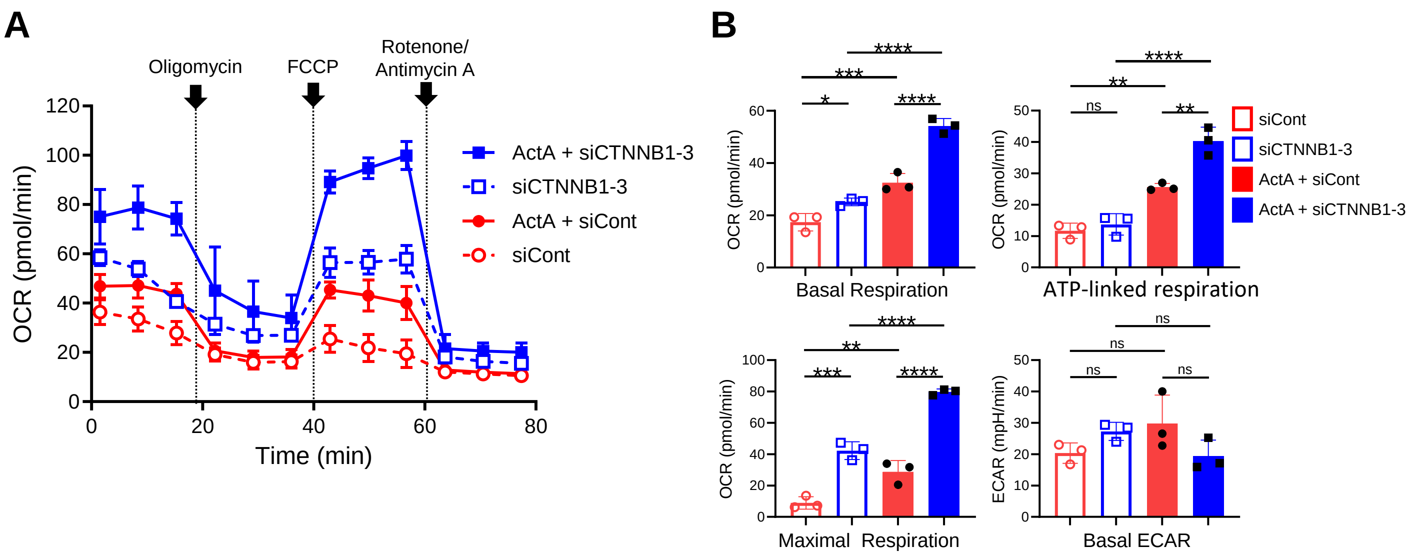

Supplementary Figure 8. Effect of suppression of β-catenin on oxidative phosphorylation (OXPHOS) in iPSCs-derived MSCs (iMSCs) on two-dimensional chondrogenic induction (2D-CI). iMSCs were transfected with siRNAs against the *CTNNB1* gene (siCTNNB1-3) or siRNA negative control (siCont) at the day before culture initiation and then cultured in chondrogenic basal medium containing with or without Activin A (ActA) (20 ng/mL) for 6 days. Subsequently, the profile of oxygen consumption rate (OCR) **(A)** and the value of each parameter **(B)** was analyzed using a Seahorse flux analyzer. Statistical significance was determined using one-way ANOVA. *$P < 0.05$, **$P < 0.01$, ***$P < 0.001$ and ****$P < 0.0001$. Values are mean ± SD. n=3, independent experiments.

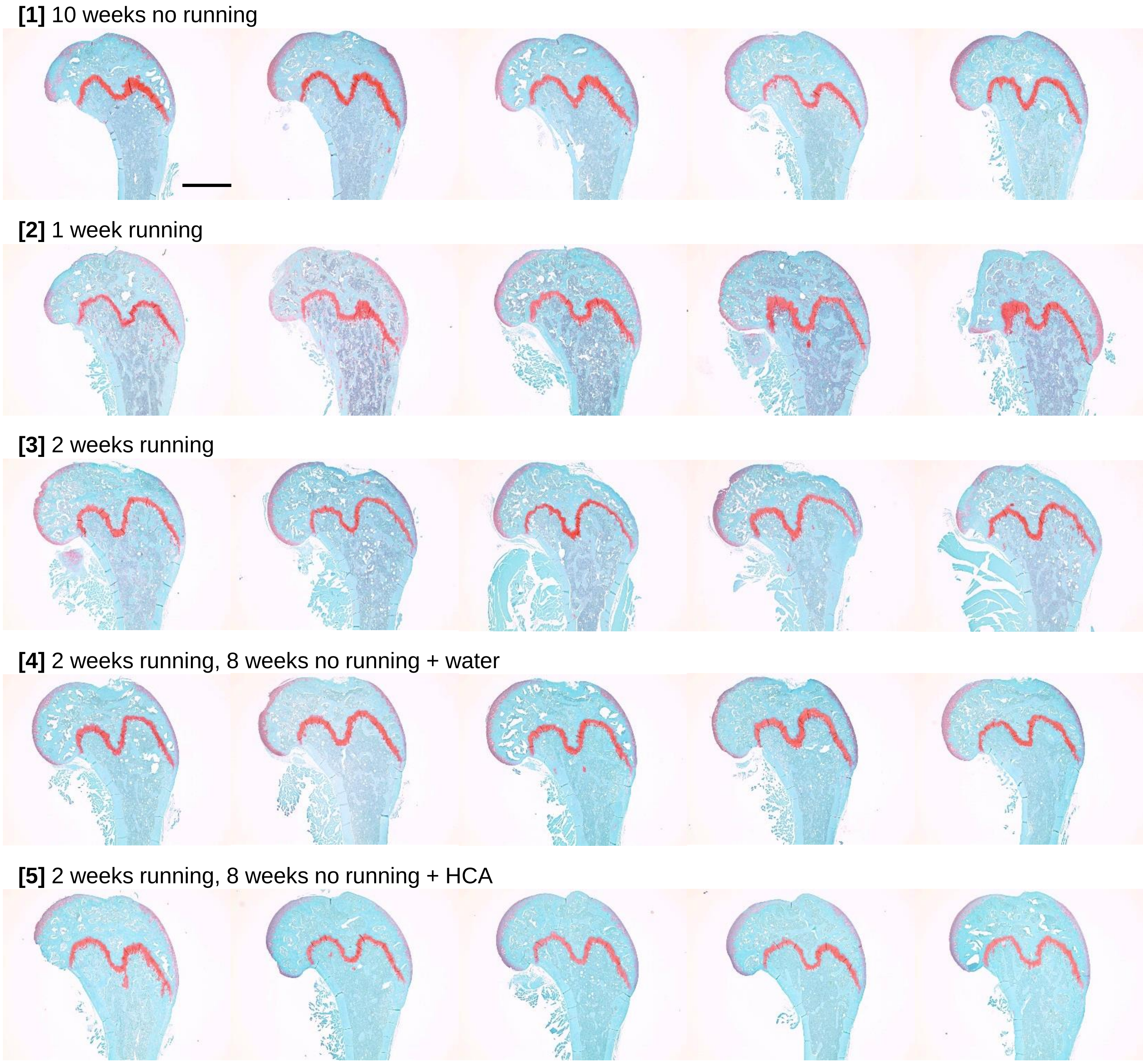

Supplementary Figure 9. Orally administered hydroxycitric acid (HCA) reconstructs damaged articular cartilage in knee joints of mice. Histological evaluation of articular cartilage. Mice from each group ([1] to [5]) were sacrificed at the end of each schedule and sections of articular cartilage from the medial condyles of femurs were stained by Safranin O. Scale bar indicates 100 µm.

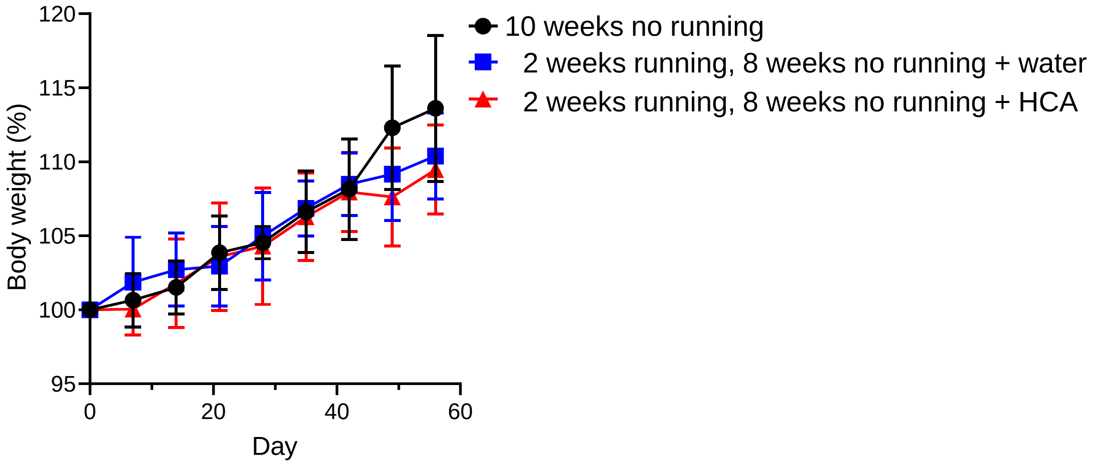

Supplementary Figure 10. Body weight changes during in vivo experiments. Body weight at each time point is shown as a value relative to those at day 0. Mean value ± SD at each point was shown (*n*= 5 in each group).

Supplementary Table S1.

A, Basal medium for iMSC

Modulator	Source and Catalog# or RRID	Solvent/Vehicle	Concentration
αMEM	Nacalai Tesque, Inc., 21444-05	Media	-
FBS	Biocera	Media	10% (v/v)
penicillin/streptomycin	Nacalai Tesque, Inc., 26253-84	Media	50units/mL penicillin, 50 μg/mL streptomycin
rhFGF2	Wako, 068-04544	0.1% BSA	5ng/mL

B, Basal medium for BM-MSC

Modulator	Source and Catalog# or RRID	Solvent/Vehicle	Concentration
αMEM-GlutaMAX	Gibco, 32571-036	Media	-
FBS	Biocera	Media	10% (v/v)
penicillin/streptomycin	Nacalai Tesque, Inc., 26253-84	Media	50units/mL penicillin, 50 μg/mL streptomycin
rhFGF2	Wako, 068-04544	0.1% BSA	2ng/mL

Supplementary Table S2.

A, Chondrogenic basal medium

Modulator	Source and Catalog# or RRID	Solvent/Vehicle	Concentration
DMEM: F12 (50:50)	Invitrogen, 11320	Media	-
FBS	Biocera	Media	0.2% (v/v)
Penicillin/Streptomycin	Nacalai Tesque, Inc., 26253-84	Media	100units/mL penicillin, 100μg/mL streptomycin
ITS premix	Corning, 354352	Media	1% (v/v)
L-ascorbic acid 2-phosphate	Sigma-Aldrich, A8960	DW	170 μM
Proline	Sigma-Aldrich, P-5607	DW	350 μM
Dexamethasone	Fujifirm Wako Pure Chemical, 047-18863	DW	100 nM
45% Glucose	Sigma-Aldrich, G8769	Media	0.15% (v/v)
Sodium pyruvate	Sigma-Aldrich, S8636	Media	1 mM
GlutaMax-I	Gibco, 35050	Media	2 mM

B, Additional reagents

Modulator	Source and Catalog# or RRID	Solvent/Vehicle	Concentration
Activin A	R&D Systems, 388-AC	4mM HCl with 0.1% BSA	20 ng/mL
TGFβ3	R&D Systems, 243-B3	4mM HCl with 0.1% BSA	5 ng/mL
BMP7	R&D Systems, 354-BP	4mM HCl with 0.1% BSA	20 ng/mL
(-)-Hydroxycitric acid calcium salt	Fujifirm Wako Pure Chemical, 084-07821	DW	100 μM (0 to 200 μM)
Dimethyl α-ketoglutarate	Fujifirm Wako Pure Chemical, OR45005	DMSO	0 to 5,000 μM
Trichostatin A	Sigma-Aldrich, T8552	DMSO	1 nM
TMRE	Invitrogen, T668	DMSO	150 nM
IACS-010759	Selleck Chemicals, S8731	DMSO	0 to 8 nM
CHIR-99021	AXON, Axon1386	DMSO	1 μM

Supplementary Table S3.

A, Primers for RT-qPCR, sequence of target gene

Target gene	Direction	Sequence
ACTB	Forward	CACCATTGGCAATGAGCGGTTC
	Reverse	AGGTCTTTGCGGATGTCCACGT
SOX9	Forward	GACTTCCGCGACGTGGAC
	Reverse	GTTGGGCGGCAGGTACTG
COL2A1	Forward	CGAGGCAACGATGGTCAGCC
	Reverse	TGGGGCCTTGTTCACCTTTGA
ACAN	Forward	TCGAGGACAGCGAGGCC
	Reverse	TCGAGGGTGTAGCGTGTAGAGA
ACLY	Forward	TGCCGACTACATCTGCAAAG
	Reverse	GGTTCAGCAAGGTCAGCTTC
P4HA1	Forward	GGCAGCCAAAGCTCTGTTAC
	Reverse	AAAGCAGTCCTCAGCCGTTA
CTNNB1	Forward	GAAACGGCTTTCAGTTGAGC
	Reverse	CTGGCCATATCCACCAGAGT

B, Product number of siRNA

Target gene	siRNA name	Product Number
ACLY	siACLY-1	s915
	siACLY-2	s916
	siACLY-3	s917
P4HA1	siP4HA1-1	s9975
	siP4HA1-2	s224153
	siP4HA1-3	s224154
CTNNB1	siCTNNB1-1	s436
	siCTNNB1-2	s437
	siCTNNB1-3	s438
NA	siCont	4390843

These siRNAs were purchased from Thermo Fisher Scientific (Waltham, MA, USA).

Supplementary Table S4. Antibodies for WB and IP

Antigen name	Source and Catalog# or RRID	Host species	Application	Dilution	Application specific details
β-Actin (13E5) Rabbit mAb (HRP Conjugate)	CST, #5125	Rabbit	WB	_1:50,000	Blocking One-P, 1hrs RT
SMAD1 (D59D7) XP® Rabbit mAb	CST, #6944	Rabbit	WB	_1:1,000	Solution 1, 1hrs RT
Phospho-SMAD1/5 (Ser463/465) (41D10) Rabbit mAb	CST, #9516	Rabbit	WB	_1:1,000	Solution 1, 1hrs RT
IDH1 (D2H1) Rabbit mAb	CST, #8137	Rabbit	WB	_1:1,000	Solution 1, 1hrs RT
IDH2 (D8E3B) Rabbit mAb	CST, #56439	Rabbit	WB	_1:1,000	Solution 1, 1hrs RT
Anti-SOX9 antibody	Abcam, ab185230	Rabbit	IP	_1:100	Binding & Washing Buffer of Dynabeads Protein G kit (Invitrogen), 10min RT
Sox9 (D8G8H) Rabbit mAb	CST, #82630	Rabbit	WB	_1:1,000	Solution 1, 1hrs RT
Acetylated-Lysine Antibody	CST, #9441	Rabbit	WB	_1:1,000	Solution 1, 1hrs RT
β-Catenin (D10A8) XP® Rabbit mAb	CST, #8480	Rabbit	WB	_1:1,000	Solution 1, 1hrs RT
Acetyl-β-Catenin (Lys49) (D7C2) Rabbit mAb	CST, #9030	Rabbit	WB	_1:1,000	Solution 1, overnight at 4 ℃
Anti-Acetyl-Histone H3 Antibody	Merck Millipore, #17-615	Rabbit	WB	_1:5,000	Solution 1, overnight at 4℃
Anti-Histone H3 Antibody, CT, pan	Merck Millipore, #07-690	Rabbit	WB	_1:5,000	Solution 1, overnight at 4℃
Anti-rabbit IgG, HRP-linked Antibody	CST, #7074	Goat	WB	_1:1,000	Solution 2, 1hrs RT

Solution 1 = 10 mM Sodium Phosphate, 150 mM Sodium Chloride, 0.1 % Sodium Azide pH 7.2
Solution 2 = 10 mM Sodium Phosphate, 150 mM Sodium Chloride, 0.1 % Sodium Azide pH 7.4
